# The Basic Immune Simulator: An agent-based model to study the interactions between innate and adaptive immunity

**DOI:** 10.1186/1742-4682-4-39

**Published:** 2007-09-27

**Authors:** Virginia A Folcik, Gary C An, Charles G Orosz

**Affiliations:** 1 Pulmonary, Allergy, Critical Care and Sleep Medicine Division, Department of Internal Medicine, The Ohio State University College of Medicine, 3102 Cramblett Hall, 456 W.10^th ^St., Columbus, Ohio, 43210, USA; 2 Divison of Trauma/Critical Care, Department of Surgery, Northwestern University Feinberg School of Medicine, 10-105 Galter Pavillion, 201 East Huron, Chicago, IL, 60611, USA; 3 Department of Surgery/Transplant, The Ohio State University College of Medicine, 350 Means Hall, 1654 Upham Dr., Columbus, Ohio, 43210, USA

## Abstract

**Background:**

We introduce the Basic Immune Simulator (BIS), an agent-based model created to study the interactions between the cells of the innate and adaptive immune system. Innate immunity, the initial host response to a pathogen, generally precedes adaptive immunity, which generates immune memory for an antigen. The BIS simulates basic cell types, mediators and antibodies, and consists of three virtual spaces representing parenchymal tissue, secondary lymphoid tissue and the lymphatic/humoral circulation. The BIS includes a Graphical User Interface (GUI) to facilitate its use as an educational and research tool.

**Results:**

The BIS was used to qualitatively examine the innate and adaptive interactions of the immune response to a viral infection. Calibration was accomplished via a parameter sweep of initial agent population size, and comparison of simulation patterns to those reported in the basic science literature. The BIS demonstrated that the degree of the initial innate response was a crucial determinant for an appropriate adaptive response. Deficiency or excess in innate immunity resulted in excessive proliferation of adaptive immune cells. Deficiency in any of the immune system components increased the probability of failure to clear the simulated viral infection.

**Conclusion:**

The behavior of the BIS matches both normal and pathological behavior patterns in a generic viral infection scenario. Thus, the BIS effectively translates mechanistic cellular and molecular knowledge regarding the innate and adaptive immune response and reproduces the immune system's complex behavioral patterns. The BIS can be used both as an educational tool to demonstrate the emergence of these patterns and as a research tool to systematically identify potential targets for more effective treatment strategies for diseases processes including hypersensitivity reactions (allergies, asthma), autoimmunity and cancer. We believe that the BIS can be a useful addition to the growing suite of in-silico platforms used as an adjunct to traditional research efforts.

## Background

The presence and effect of biocomplexity on biomedical research is well recognized [1-7]. As a result, there is rapidly growing interest in the development of "in-silico" research tools to be used as an adjunct to more traditional research endeavors [8-14]. The host response to insult is one of the most striking examples of biocomplexity [7,15]. The innate immune response is essential for immunity to bacterial, fungal and parasitic infections. The cells of the innate immune system recognize well conserved "danger" signals [16], and innate immunity was the first part of the immune system to evolve [17]. The basic strategy of innate immunity is to kill and clear pathogens. The innate immune system is also recognized to contribute to the pathophysiology of such wide-ranging diseases as atherosclerosis, lung fibrosis, asthma and sepsis [17,18]. The adaptive immune response, which follows the innate response, is responsible for fighting disease and developing into the memory response. This process involves exponential proliferation of antigen-specific cells that rapidly eliminate pathogens upon a second encounter. Adaptive immunity is also responsible for processes such as hypersensitivity reactions, autoimmune diseases, cancer and transplant rejection. Both the innate and adaptive components of the host response are complex, and the interaction between the two represents another level of intricate, non-linear and potentially paradoxical behavior [7,16,19]. In order to aid in the qualitative characterization and examination of this relationship, we introduce the BIS, an agent-based model (ABM) based on the cellular and molecular mechanisms of the interface between the innate and adaptive immune response.

Agent-based modeling has been used to study the non-linear [6] behavior of complex systems [20,21]. This technique is also known as "individual-based modeling", "bottom-up modeling" [20] and "pattern-oriented modeling" [22]. Agents and signals are used to represent the basic elements of a complex system, and the agents interact with each other in a computer-simulated environment. While the goal was to represent all of the basic types of cells that populate the immune system in the model, we did not attempt to replicate every known sub-type of immune cell (Table 1). This abstraction is a necessary step in the translation of real-world systems to mathematical or simulation models, and is targeted at the coarsest level of granularity that can effectively reproduce the behavior of the overall system at a pre-specified level of interest [22]. For purposes of the BIS we have chosen to focus primarily at the "cell-as-agent" level of resolution. Our rationale for this is that cells represent a well-defined biological organizational level, and that extensive information exists regarding the behaviors of cellular populations in response to extracellular stimuli. We believe that cells can be treated as finite state machines that can be readily grouped into classes that would correspond to agent-classes sharing the same behavioral rules.

**Table 1:** Summary of the agents, signals and behaviors in the Basic Immune Simulator.

AGENT TYPES AND ZONES	IMMUNE CELLS REPRESENTED AND FUNCTIONAL DESCRIPTION	SIGNALS	CYTOKINES, CHEMOKINES [28] AND MOLECULES REPRESENTED BY EACH SIGNAL
Parenchymal Cell Agent (PC) Zone 1	Functional tissue cells	(Parenchymal-kine 1) PK1	Stress factors such as Heat Shock Proteins [66], Uric Acid [67], and Chemerin [68], chemokines such as CX3CL1, CCL3, CCL5, CCL6
		Virus	Virus particles
		Apoptotic bodies	Apoptotic bodies or dead cells associated with programmed cell death
		Necrosis factors	Cell fragments associated with death by necrosis
Dendritic Cell Agent (DC1, DC2) Zones 1, 2	Tissue surveillance, antigen presentation, INNATE immunity	(Mono-kine 1) MK1	IL-12, IL-8 (CXCL8) [69], CCL3, CCL4, CCL5, CXCL9, CXCL10, CXCL11
		(Mono-kine 2) MK2	IL-10, CCL1, CCL17, CCL22, CCL11, CCL24, CCL26
Macrophage Agent (MΦ1, MΦ2)Zone 1	Scavenging of dead cell debris, antigen presentation, INNATE immunity	MK1	IL-12, IL-8 (CXCL8), CCL3, CCL4, CCL5, CXCL9, CXCL10, CXCL11
		MK2	IL-10, CCL1, CCL17, CCL22, CCL11, CCL24, CCL26
T Cell Agent (T, T1, T2) Zones 2,3,1	T _HELPER _lymphocytes, cell-mediated, ADAPTIVE immunity	(Cytokine 1) CK1	IFN-γ, IL-2, TNF-β
		(Cytokine 2) CK2	TGF-β, IL-4, IL-5, IL-6, IL-10, IL-13
Cytotoxic T Lymphocyte Agent (CTL) Zones 2,3,1	T _CYTOTOXIC _lymphocytes, cell-mediated, ADAPTIVE immunity	CK1	IFN-γ
Natural Killer Cell Agent (NK) Zone 1	Natural Killer Cells, cell-mediated immunity, kills stressed cells, INNATE immunity	CK1	IFN-γ
B Cell Agent (B, B1, B2) Zones 2,3,1	B Lymphocytes, ADAPTIVE, humoral immunity, makes antibodies	(Antibody 1) Ab1	Cytotoxic and neutralizing antibody
		(Antibody 2) Ab2	Targeting and neutralizing antibody
		Complement	Bound antibody catalyzes complement product formation, C3a, C5a [70]
Granulocyte Agent (Gran) Zones 3,1	Neutrophils, Eosinophils and Basophils, INNATE immunity, releases enzymes and toxins by degranulation and produces reactive oxygen species	(Degranulation product 1) G1	Degranulation products, reactive oxygen products
Portal Agent Zones 1,2,3	Blood vessels, lymphatic ducts. The only agent representing a structure rather than a cell type.		

All of the programmed entities that exist in the simulation are listed in the "AGENT TYPES AND ZONES" and "SIGNALS" columns. The words "cell" or "lymphocyte" are meant to refer to the actual living structure. The word "agent" refers to the representation or programmed object that exists in the simulation.

One example of abstraction in the model is the representation of cytokines and chemokines with simulated signals that fall into two categories: signals that up-regulate the response (type 1) and signals that down-regulate the immune response (type 2). For the T Cell agents (Ts), the cytokine-1 (CK1) and cytokine-2 (CK2) signals represent *all *of the cytokines and chemokines produced by T_HELPER_-1 and T_HELPER_-2 lymphocytes, respectively. Table 1 lists the simulated signals within the model and the cytokines/chemokines that they are intended to represent. These are not meant to be exhaustive lists.

Table 2 lists the behaviors for all of the cellular agents participating in the simulation. Behaviors have been defined as interactions between the agent and the environment, the latter including other agents. Intracellular signal transduction events are considered to be implied in the agent's state (another example of abstraction in the model, as mentioned above). Each agent detects signals and other agents, and responds to them in a way that is dependent upon their current state. The details for these behavioral rules for all of the agents are represented as *state diagrams *[see Additional files 1, 2, 3, 4, 5, 6, 7, 8, 9, 10, 11, 12, 13, 14, 15, 16]. Table 2 is also a reference list for the basis of the rules.

**Table 2:** Summary of literature citations for agent behaviors

**Agent types**	**Behaviors**	**Citations**
Parenchymal agent (PC)	Signal production	[66-68]
	Neighbor detection/contact/killing	[71]
	Migration	Not applicable (NA)
	Proliferation	NA
	Death	[55, 70, 72-75]
Dendritic Cell agent (DC)	Signal detection	[38, 76-79]
	Signal production	[38, 77, 80, 81]
	Neighbor detection/contact/killing	[30, 31, 38, 80-89]
	Migration	[28, 30, 38]
	Proliferation	[38]
	Death	[57, 83, 89-91]
Macrophage agent (MΦ)	Signal detection	[16, 17, 66, 70, 73, 92]
	Signal production	[92]
	Neighbor detection/contact/killing	[55, 73, 75, 85]
	Migration	[28, 66, 70]
	Proliferation	NA
	Death	[93]
T Cell agent (T)	Signal detection	[38, 81]
	Signal production	[81, 85]
	Neighbor detection/contact/killing	[30, 36, 38, 81, 83, 86, 94, 95]
	Migration	[28, 30]
	Proliferation	[83, 95]
	Death	[71, 91, 96]
Cytotoxic T Lymphocyte agent (CTL)	Signal detection	[97]
	Signal production	[87, 98]
	Neighbor detection/contact/killing	[87, 97, 98]
	Migration	[28]
	Proliferation	[97]
	Death	[99]
Natural Killer agent (NK)	Signal detection	[66, 72, 100]
	Signal production	[101]
	Neighbor detection/contact/killing	[86, 99, 100, 102]
	Migration	[28]
	Proliferation	NA
	Death	[99]
B Cell agent (B)	Signal detection	[82, 103]
	Signal production	[73, 82, 103, 104]
	Neighbor detection/contact	[36, 82, 103]
	Migration	[28, 104]
	Proliferation	[36, 103, 105]
	Death	[82, 103]
Granulocyte agent (Gran)	Signal detection	[28, 70]
	Signal production	[74]
	Neighbor detection/contact/killing	[55, 74]
	Migration	[28]
	Proliferation	[74]
	Death	[74]
Portal Agent (Portal)	Signal detection	[28]
	Signal production	[28]
	Neighbor detection/contact/killing	[28]
	Migration	[28]
	Proliferation	NA
	Death	[55]

The BIS is intended to take the abundance of information available in the immunology literature, condense it into logical rules for the agents participating in a simulated immune response, and instantiate the rules such that the consequences of those rules can be observed for the system as a whole [23]. In so doing the BIS attempts to address some of the limitations of the linear reductionist approach that has dominated the scientific method over the past 500 years. An integrative approach to immunology, a.k.a. *in silico *biology [3] is necessary to deal with the ongoing explosion of information generated in biomedical research, and the BIS is our contribution to the growing suite of in-silico tools.

## Implementation

### Simulation development

The BIS [24] was created using the Recursive Porus Agent Simulation Toolkit (RepastJ) library, an open-source software library that is available online [25,26].

The computer program was written with separate Java Classes for each of the agents of the BIS. The program is described in state diagrams, presented in Additional files 1, 2, 3, 4, 5, 6, 7, 8, 9, 10, 11, 12, 13, 14, 15, 16. These diagrams form a bridge between the Java computer program and the logically stated rules for behavior of the agents in the simulation. The agent behavioral rules are drawn from the immunology literature (Table 2). Separate state diagrams describe the behavior of each type of agent in each zone of the simulation that it may occupy. Agent states are determined by the values of the agent's internal (class) variables. Agent behaviors are represented by state changes in reaction to the environment, consistent with the concept of "model-based reflex agents" or "reflex agents with state" [27]. Agent rules are expressed as logical statements that represent, in an abstract manner, the intracellular processes affected by the engagement of cell-surface receptors with ligands present in the immediate environment of a living cell. Therefore, the behavior of an agent is determined by its individual local environment, allowing for heterogeneous behavior within a population of agents that share the same rules. The dynamics of the overall system is a product of the interactions of the populations of agents.

### Simulation zones

**Figure 1:**
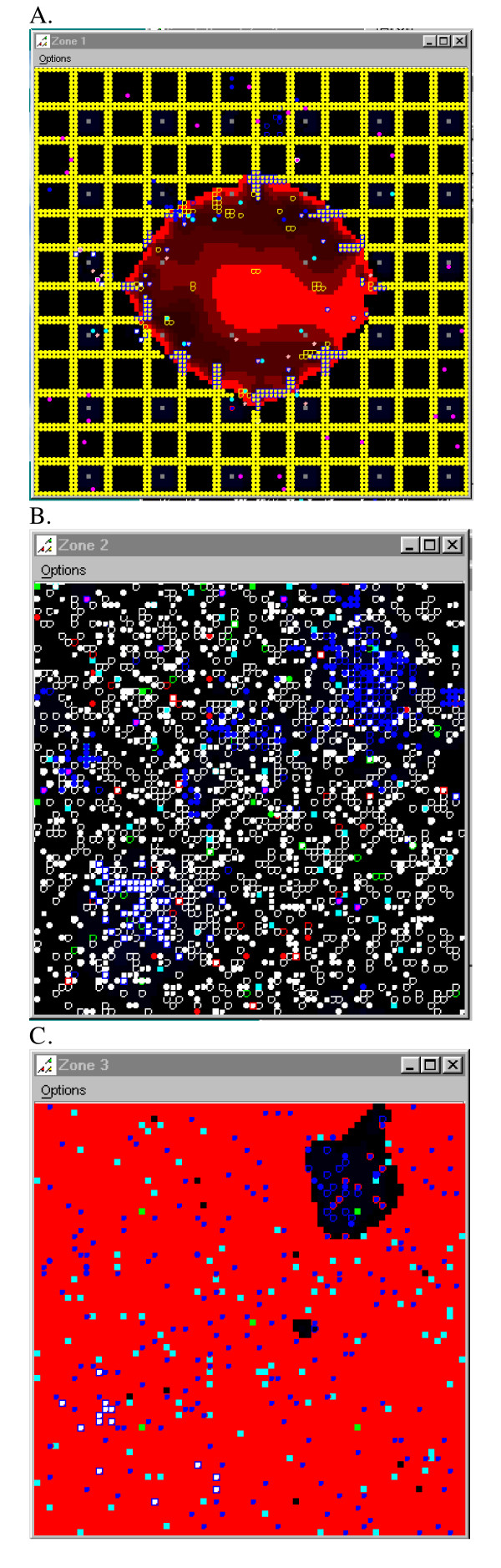
**Description of the three zones of activity of the Basic Immune Simulator**. **1a Zone 1, the parenchymal tissue zone**. This represents a generic functional tissue (yellow circles represent Parenchymal Cell agents) within the body that becomes infected with a virus (represented as the red, diffusing signal). If one assumes the average diameter of a cell to be approximately 0.01 mm, then Zone 1 represents an area of about 1.0 mm^2 ^of tissue. **1b Zone 2, the secondary lymphoid tissue zone**. Secondary lymphoid tissue includes the lymph nodes and spleen. This is the site where the agents representing the lymphoid cells (B Cell agents, T Cell agents, and Cytotoxic T Lymphocyte agents) reside, and the site where the agents representing antigen presenting cells (Dendritic Cell agents) interact with the lymphoid agents causing them to proliferate. **1c Zone 3, the blood and lymphatic circulation**. When the agents in the secondary lymphoid tissue proliferate (Zone 2), they migrate into the lymph/blood (Zone 3) and then travel back to the initial infection site (Zone 1).

The BIS was created with three "zones" of activity to represent the separate locations in the body where interactions between cells take place during the course of an immune response (Figure 1). Zone 1 is the site of initial tissue challenge with pathogen. In this model of viral infection Zone 1 represents a generic parenchymal tissue. Zone 1 also contains resident Dendritic Cell agents (DCs). Zone 2 is an abstract representation of a lymph node or the spleen, where lymphocytes reside and proliferate. Zone 3 is an abstract representation of the lymphatic and blood circulation, the conduits for travel for the cells of the immune system. Zone 3 was created to contain the agents that represent cells that must travel for indefinite (unknown) periods of time before arriving at the final destination, the site of pathological challenge (Zone 1). Thus Zone 3 can be considered the "rest of the body" and circulation apart from the areas of actual infection (Zone 1) and the areas of immune cell proliferation (Zone 2). The agents that represent lymphocytes that have proliferated in Zone 2 and the Granulocyte agents are the agent types found in Zone 3. The Portal agents (Portals) in Zone 3 representing spatially discreet blood and lymphatic vessels control the access of the agents to Zone 1. They also transmit signals produced in Zones 1 and 2 to attract agents to migrate. The Portals also participate in the transport of some signals to Zone 1. Portals are a means of transferring agents and signals from one zone to another. They are randomly placed in Zones 2 and 3. The variation and uncertainty of the time spent by immune cells in the areas represented by Zone 3 is one of the sources of randomness in the BIS.

The graphical representations of the zones are shown in Figures 1a–1c. The zones are two-dimensional toroidal grids that allow for the presence of more than one agent or signal at any (x, y) coordinate in the grid. The dimensions of the grids are set by the input parameters: World1XSize, World1YSize, etc. [see Additional file 17]. The sizes remained constant for all of the experiments presented. The dimensions of the zones represent microscopic areas of tissue for Zone 1 and Zone 2, with enough area for the necessary interactions to take place. This is an abstraction of a localized infection, with draining lymph nodes participating in the immune response. Minimal zone sizes were selected that would allow one to observe the interactions and still have a simulation that would be able to run on the average personal computer. All of the other numbers of agents were chosen to be in proportion with what was already implemented and to resemble cell proportions in living systems as well as possible. As agents were "programmed into" the simulation, their numbers were adjusted until there were enough of them to participate in a simulation run, and engage in the desired behavior patterns [see Additional files 1, 2, 3, 4, 5, 6, 7, 8, 9, 10, 11, 12, 13, 14, 15, 16]. Many quantities are unknown for living systems, because measurements are either static (require sacrificing a mouse and getting one time point) or indirect (measured in the blood). One has to try to create the simplest possible representation, and still capture the patterns of behavior that one wants to study. This requires incrementally adjusting quantities of agents and signals until the desired pattern(s) appear.

Lymphocytes and the cells of the innate immune system follow chemokines generated in response to a pathological challenge [28]. Agents will "follow" a gradient toward a higher concentration if the relevant signal (representing a chemotactic mediator) is present. When any agent is in motion, it may only move to one of its eight adjacent grid spaces (its Moore Neighborhood). Agents are also capable of moving from one zone to another, simulating the trafficking of immune cells from one tissue type to another.

### Simulation progression of events

The simulation progresses in discrete intervals called "ticks". This mechanism simulates concurrency [29], and provides a qualitative sequential representation of the events that occur in an immune response. At each tick each agent executes its rule sequence, probing its immediately adjacent locations and reacting to the information that it detects. All information about quantities of agents of each type and quantities of signal is recorded for each zone at the end of every tick.

Events such as dendritic cell tissue surveillance and response to a pathogen, antigen presentation to lymphocytes, and circulatory transport time, incorporate a stochastic component in the form of random motion of the agents (when not influenced by chemotactic signals). This is consistent with the recorded random motion of fluorescently labeled dendritic cells and T lymphocytes in murine lymph nodes [30]. Additionally, naïve T-lymphocytes move randomly from lymph node to lymph node throughout the body to increase their probability of encountering the antigen that they recognize on an antigen presenting cell in any particular lymph node [28].

In general, the agents probe their Moore Neighborhood with a radius of one space. The only exception are DCs, which probe a radius of two grid spaces, for a surrounding total of twenty-four grid spaces. This is to reflect the highly developed ability of dendritic cells to probe their surrounding environment [31]. Information about agents and signals within a probed zone constitutes the local environment for a particular agent, and subsequently affects its behavior and state changes.

### Simulation agents

Agents represent the cells of the immune system, the parts of the lymphatic and circulatory system that allow immune cells to migrate, and the functional (parenchymal) cells of a generic tissue. For the complete list see Table 1. Each agent type executes behaviors that are summarized with references in Table 2. The details of the rules for behavior of all of the agents are presented in Additional files 1, 2, 3, 4, 5, 6, 7, 8, 9, 10, 11, 12, 13, 14, 15, 16 with state diagrams.

The agents representing the cells of innate immunity, the DCs, Macrophage agents (MΦs) and Natural Killer agent (NKs), are cells generally believed to be produced as precursors in the bone marrow, and circulate in the blood at levels maintained by undefined mechanisms [32]. These agents enter Zone 1, the simulated parenchymal tissue via portals in response to "danger signals" [16,17]. The conditions that cause entry are written in magenta in the state diagram of the DCs [see Additional file 3]. The quantities of these agents that enter are in the green boxes that signify input parameters (numXToSend).

The agents representing the cells of adaptive immunity, the B Cell (B), T Cell (T) and CTL agents (CTLs), proliferate in response to contacts with DCs and each other in Zone 2 (the lymph node). The proliferation mechanisms are in the state diagrams [Additional files 1, 2, 3, 4, 5, 6, 7, 8, 9, 10, 11, 12, 13, 14, 15, 16, in magenta] for these agents, and the green boxes have the input value numXToSend that indicates how many more of the agents will be added to Zone 2. When these agent types proliferate, their progeny are created and placed in the zone within the Moore Neighborhood of where the original agent resides.

All of the agent types have input parameters that pre-determine their "lifetimes", and these parameters were kept constant for all of the experiments presented. All agents may (stochastically) experience events that shorten (or lengthen) their lifetimes, and these rules override the input parameters.

### Signal diffusion

At the beginning of each tick, all of the signals "diffuse" through the zones that contain them. Any addition of signal (by an agent) from the previous tick occurs at this time. The simulated diffusion is an abstraction of the cytokine and chemokine release and diffusion process. The diffusion process is implemented as follows for each matrix location in a zone:

New value = evaporation rate (current value + diffusion constant (nghAvg - current value))

See the Repast Javadoc, class Diffuse2D, method diffuse() [25] for details. The evaporation rate (evapRate) and the diffusion constant (diffusionConstant) are input parameters [see Additional file 17] and nghAvg is a weighted average of the values for a signal in the location's Moore Neighborhood. The "New value" and "current value" are local variables. The signal gradients generated by the diffusion process simulate the chemotactic gradients that affect cellular movement. All signals in the simulation use the same diffusion rate parameters. This abstraction is necessary because the rates of diffusion of cytokines and chemokines in living tissue are unknown.

### Simulation validation and testing

The starting values for the variables [see Additional file 17] were determined by preliminary experiments conducted during the development of the simulator and refined via an iterative process. An input parameter sweep was performed to identify patterns of BIS behavior that matched patterns of normal behavior observed in living systems. This is a pattern-oriented analysis procedure termed "indirect parameterization" by Grimm and Railsback [29]. Since the goal was to study the immune system fighting disease, the default values for all of the parameters were chosen to allow the immune system agents to participate in eliminating the simulated infection in the majority of simulation test runs. For some of the agent types, it was possible to find **estimates **of the numbers of the represented cell types that would be found in tissue [33-35]. Some input parameters were never changed, but were included in Additional file 17 for documentation purposes.

We verified the behavior of the agents, i.e. ensured that the agents were behaving as intended, as reflected in their state diagrams [Additional files 1, 2, 3, 4, 5, 6, 7, 8, 9, 10, 11, 12, 13, 14, 15, 16] by repeatedly having randomly selected individual agents produce (printed) output demonstrating state changes during the course of a run. The signals and neighboring cells that they detected that caused their state changes were also recorded. All agent types were programmed to produce output that indicated that all of the lines of the computer program were executed under the proper conditions. All executable behaviors in all agent types were tested.

### Simulation experiments and data generation

Initial conditions for each experimental run included the scenario (Set_ViralInfection), cell population numbers(PercentXAntiViral, NumXToSend, NumDendriticAgents, NumGranZ3denom, PercentProInflammatory) and signal strengths (IncrementOutputSignal, OutputSignal; see Additional file 17). Default values for all of the initial conditions were programmed into the simulation; deviations from these default values represented the variation of initial input. It is only necessary to enter the values (via the GUI or a batch text file) that will differ from the default values. The initial conditions are recorded and the output data is collected from all of the simulation runs and saved in text files.

Variations in BIS behavior between the simulation runs within an experimental set results from stochasticity built into the model. The sources of random variation built into the model are: 1. Initial agent placement, except for PCs, 2. Random motion and Zone 3 delay, and 3. Stochastic effects on agent "lifetime" (discussed above). While the initial conditions for the numbers and types of agents in every zone are constant for a set of experiments, the random placement of some of the agents is accomplished using a random number generator to choose the (x, y) coordinates for their location. Another source of variation is the amount of time agents spent in Zone 3, the representation of the lymphatics/blood. These sources of randomness were enough to make every run of the BIS unique.

Of note, not all sets of initial experimental conditions were run the same number of iterations. This was because some runs ended with the "immune hyper-response", halting the progression of the batch runs by exhausting the random access memory of the computer. We identified this effect to be due to exponentially increasing numbers of lymphocyte agents due to forward feedback. Despite this behavior, the validity of the *immune hyper-response *outcome is discussed in greater detail in the Results and Discussion.

## Results and discussion

### Simulation outcomes with various initial conditions

Initial parameter sweeps of the BIS identified three outcome patterns. The first, when the simulated immune system eliminated the virally infected PCs and allowed regeneration to take place, is called an "immune win". Second, when the simulated immune system failed to eliminate the virally infected PCs and all of the PCs became infected or the majority of the tissue failed to regenerate is called an "immune loss". Both of these outcomes were expected. However, the third pattern was less intuitive and involved positive feedback behavior that resulted in the proliferation of agents representing lymphocytes in Zone 2, exhausting the computer memory available for the simulation (Table 3). This outcome was considered an "immune hyper-response". Exponential lymphocyte proliferation is normal behavior in response to antigen-specific presentation events in the lymph node, and it is necessary for generation of sufficient numbers of lymphocytes to fight infection and generate memory cells [36]. Under normal conditions, various mechanisms exist (including removal of stimulus, *i.e. *resolution of infection) to put an end to the proliferation. Rather than trying to correct the program, this outcome was regarded as legitimate and considered to represent a "hypersensitivity" pattern. Hypersensitivity reactions are recognized in various disease states, and they involve excessive pathological contribution from the lymphocytes that these agent types represent [37].

**Table 3:** Initial conditions and agent types involved in the immune hyper-response

**Initial conditions**	**Fraction of simulation runs with identical starting conditions that ended in the *immune hyper-response *due to the agent types given.**					
(Conditions in Figure 2)	**T1**	**T2**	**B1**	**B2**	**CTL**	**Number of runs**

**10 DCs**	0.07	0.93	0.07	0.29	0	14
**20 DCs**	0	0.89	0.22	0.22	0	9
**30 DCs**	0.38	0.75	0.25	0.25	0	8
**40 DCs**	0.57	0.57	0.43	0.29	0	7
**50 DCs**	0	1.0	0	0	0	1
**60 DCs**	0.93	0.29	0.93	0	0	14
**70 DCs**	0.89	0.26	0.89	0.05	0	19
**80 DCs**	1.0	0.25	1.0	0	0	4
**90 DCs**	1.0	0.12	0.75	0.12	0	8
**100 DCs**	1.0	0.18	1.0	0.09	0	11
**No DC apoptosis**						
**50 DCs**	0.86	0.09	0.32	0.03	0.11	66
**Exclusion of CTLs from the simulated immune response**						
**20 DCs**	0.17	0.83	0.25	0	0	12
**50 DCs**	0.83	0.33	0.83	0	0	6
**80 DCs**	0.86	0.57	0.86	0.14	0	7
**Exclusion of NKs from the simulated immune response**						
**20 DCs**	0.09	0.91	0.09	0.36	0	22
**50 DCs**	0.50	0.67	0.46	0.29	0.04	24
**80 DCs**	0.79	0.38	0.69	0.14	0	29
**Exclusion of MΦs from the simulated immune response**						
**20 DCs**	0.10	0.84	0.10	0.16	0	19
**50 DCs**	0.64	0.45	0.64	0.09	0	11
**80 DCs**	1.0	0	1.0	0	0	1
**More DCs recruited at immune activation**						
**20 DCs**	0.50	0.22	0.28	0	0.02	40
**50 DCs**	0.68	0.25	0.54	0.04	0.11	28
**80 DCs**	0.87	0.17	0.74	0.09	0.13	23
**Increased CTL proliferation at activation**						
**20 DCs**	0.10	0.30	0.10	0	0.70	10
**50 DCs**	0.26	0.17	0.35	0	0.87	23
**80 DCs**	0.50	0	0.50	0	1.0	4

For the simulation runs that ended in the *immune hyper-response*, the fraction of the runs in which the agents representing the lymphocytes in Zone 2 that proliferated excessively are given. The fractions do not add up to 1.0 for each row because more than one agent type may have proliferated excessively. The agent types were counted as contributing to the hyper-response if more than 900 agents were present in Zone 2 at the time when the simulation run terminated. The simulation was programmed to terminate when more than 30,000 agents were detected to be participating at a given time. There were multiple check points to count the number of agents participating.

This is intended to be a qualitative model, and as such the goal is to reproduce "recognizable" patterns of behavior seen in biological systems. The model effects that come from the model implementation result from the behaviors observed for the individual agents and the system. The behavior of the agents is "imposed behavior" [29]. It is the behavior programmed into the individual agents and presented in the state diagrams [see Additional files 1, 2, 3, 4, 5, 6, 7, 8, 9, 10, 11, 12, 13, 14, 15, 16]. This includes the numbers and types of agents used. The system behavior results from the complex interactions of the individual agents in the system, and this includes the *immune win*, *immune loss *and *immune hyper-response *patterns. All three of these patterns represent behavior of the real system. Since the *immune win *and *immune loss *were expected patterns, we do not consider them to be "emergent" [29]. The *immune win *could be considered imposed behavior, because this was the system pattern sought in the building of the simulation. The *immune loss *was a default pattern that occurred until a substantial portion of the BIS was completed. The *immune hyper-response *was emergent, because it was unexpected but recognized as a pattern present in the real system. In this sense we feel we have succeeded in our goal, because the behavior observed for the BIS is like that of a (human or murine) immune system.

### Simulation results from experiments varying the initial number of DCs

**Figure 2:**
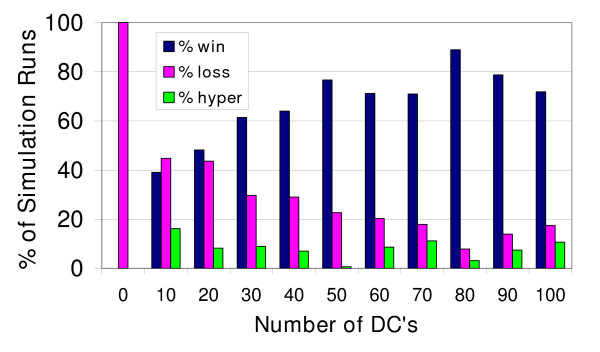
**The effect of varying the number of DCs at initialization on the immune response**. The percent of simulation runs for which the immune system eliminated the virally infected parenchymal cell agents (% win), the percent of simulation runs that ended with infection of all of the parenchymal cell agents (% loss) and the percent of simulation runs that ended with hyper-proliferation of T Cell and B Cell agents (% hyper) are shown. The number of simulation runs for each condition were as follows: 0 DC, n = 100; 10 DCs, n = 105; 20 DCs, n = 110; 30 DCs, n = 101; 40 DCs, n = 100; 50 DCs, n = 150; 60 DCs, n = 163; 70 DCs, n = 179; 80 DCs, n = 127; 90 DCs, n = 108; and 100 DCs, n = 103.

As the immunological "first responders" in tissue to a pathological challenge [38], it was expected that the initial number of DCs would significantly affect the simulated immune response. The results from experiments in which the initial number of DCs was varied are shown in Figure 2. In the absence of DCs there was 100% *immune loss*. Incremental increases in the number of DCs allowed *immune wins *to occur with a higher probability, up to a point. There is a plateau in the effect of increasing the number of DCs on the frequency of immune wins. More than 80 DCs present initially did not improve the *immune win *outcome frequency. The positive association was significant overall (Pearsons product moment correlation r^2 ^= 0.6689, p = 0.0021). The most important aspect of this result is not the actual number of DCs that had the highest probability of resulting in *immune win*, since this quantitative value is dependent upon all of the other initial conditions' values and the design of the BIS. What matters is that there is a qualitative reproduction of the outcome patterns (*immune wins *and *losses*) for the number of DCs present for surveillance. This parameter sweep of the initial number of DCs demonstrates that there is a suboptimal range of initial values for the number of DCs, there is an optimal range of values, and there is a threshold number beyond which increasing the number of DCs does not confer a benefit. Such patterns are common to biological systems.

At the same time, *immune losses *occurred with higher frequency when fewer DCs were present initially. The negative association was significant (r^2 ^= 0.6407, p = 0.0031). The frequency of the *immune hyper-response *was not correlated with the number of DCs present at initialization (r^2 ^= 0.0035, p = 0.8631). A Chi-squared contingency analysis found the ratios of outcomes (*win*, *lose*, *hyper*) to be significantly different overall among the different DC number initial condition groups (p < 0.0001).

In the cases when the simulation run ended with the *immune hyper-response*, the types of agents that proliferated excessively in Zone 2 were determined. Table 3 presents the fractions of the simulation runs that were ended by each lymphocyte agent type. When fewer than 50 DCs were present at initialization, the Type 2 response predominated. T_HELPER_-2 lymphocytes are the main adaptive immune cell type responsible for the pathology of allergies and asthma [39,40] and the initial phase of atopic dermatitis [41]. Dendritic cells are thought to be responsible for this skewing of the immune response in asthma [18]. One could speculate that the "hygiene hypothesis" [42,43] might be a real-world correlate to this observation. Exposure to microbes may be necessary to create a mature immune system with sufficient dendritic cells.

When more than 50 DCs were present, the Type 1 response progressively dominated. The lymphocytes that these agents represent are the ones that mediate damage associated with psoriasis and the secondary phase of type IV hypersensitivity reactions such as atopic dermatitis. Interestingly, inflammatory dendritic epidermal cells and increases in their recruitment have been shown to induce the pro-inflammatory adaptive immune response in these diseases [41,44].

Mice that lack myeloid dendritic cells (the in vivo correlate of DC1s) due to an integrated transgene (relB-/-) are abnormal and short-lived. They exhibit abnormal inflammation in several organs, splenomegaly, myeloid hyperplasia, a lack of normal lymph nodes (lymphocytes are present but scattered) and few thymic dendritic cells [45-47]. These mice also develop skin lesions with numerous T_HELPER_-2 cells, dramatically increased interleukin-4 (IL-4) and IL-5 and numerous eosinophils similar to human allergic atopic dermatitis. They also exhibit characteristics of allergic lung inflammation [48]. RelB-/- mice are also unable to eliminate vaccinia virus infection of the skin [49]. Such patterns are comparable to the outcome patterns of the BIS with the lowest numbers of DCs starting conditions (10 DCs), where the immune losses were highest, the immune hyper-response occurred frequently and it was T2-biased. The dendritic cells that remain in the RelB-/- mice' systems would be comparable to the DC2 population in the simulation.

### Simulation experiments with individual agent types eliminated from the immune response

**Figure 3:**
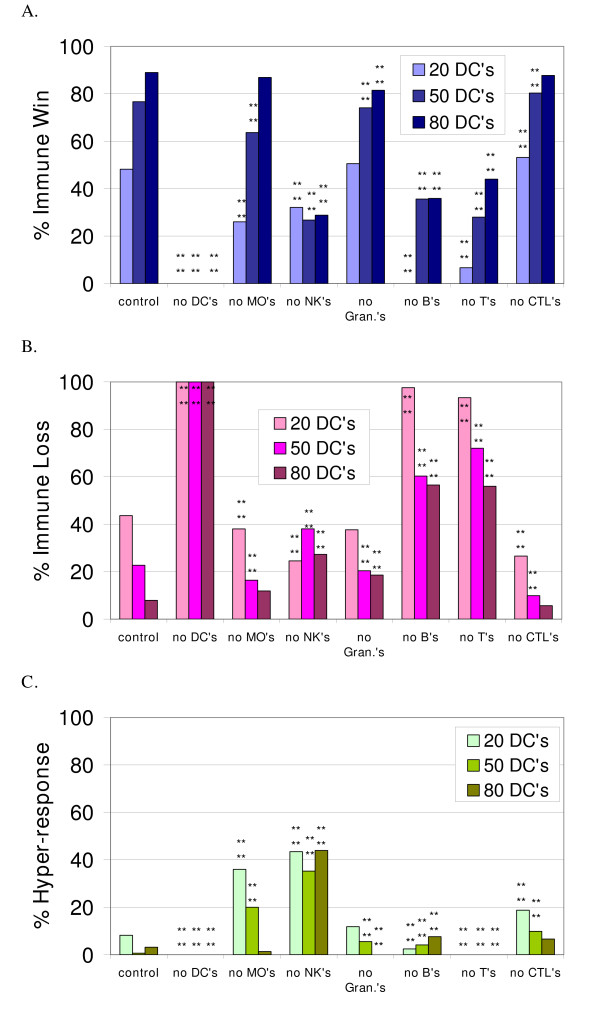
**The effect of eliminating each agent type from the simulated immune response at initialization**. Figures 3a, 3b and 3c show the percent of simulation runs that ended with the *immune win, loss and hyper-response *outcomes, respectively, when the indicated agent type was missing, in combination with initial conditions of 20, 50 or 80 DCs. The control has all cell types present. The number of simulation runs for each data bar is as follows: No Bs with 20 DCs, n = 82; with 50 DCs, n = 73; with 80 DCs, n = 92; no CTLs with 20 DCs, n = 64; with 50 DCs, n = 61; with 80 DCs, n = 106; no DCs, n = 100; no MΦs with 20 DCs, n = 50; with 50 DCs, n = 55; with 80 DCs, n = 76; no NKs with 20 DCs, n = 53; with 50 DCs, n = 71; with 80 DCs, n = 66; no Ts with 20 DCs, n = 75; with 50 DCs, n = 50; with 80 DCs, n = 50; no Granulocyte agents with 20 DCs, n = 93; with 50 DCs, n = 54; with 80 DCs, n = 54. The asterisks indicate significant differences from the control conditions using the Chi-squared test. The p-value for the bars marked **** is p <= 0.0001.

The effect of removal of each of the immune cell agent types on the success of the simulated immune response is shown in Figure 3a. These simulation runs correspond to "knock-out" in vivo experimental preparations. These simulations were performed with starting conditions of 20 DCs, 50 DCs and 80 DCs, a representative range of numbers of DCs. The frequency of the outcomes for each condition was compared to the control with the same number of DCs using a Chi-Squared Test with 2 degrees of freedom. Asterisks marking significant differences indicate that at least two of the three frequency values (*immune win*, *immune loss *or *immune hyper-response*) were different from the control. The P-values are given in the figure legend. The elimination of the DCs, Ts, Bs and NKs had the most detrimental effect on the simulated immune response. The decrease in *immune wins *with removal of each of the agent types was greater when there were fewer DCs present as well. Figure 3c shows the incidence of the *immune hyper-response*. It is interesting that the *immune hyper-response *occurred more frequently when the agent types representing the cells of the innate immune system were decreased, i.e. when the MΦs or NKs were eliminated (Figure 3c). In Table 3, the fraction of runs in which each agent type contributed to this outcome are given.

The creation of mice with specific knockout of NK cells has been very difficult, and mice without NK cells are missing other cell types as well [50], so results from those mice cannot be compared to the results described above. Suppression of NK cell function has been implicated in the pathogenesis of allergies [51] and the exacerbation of experimental autoimmune encephalomyelitis [52]. Both are abnormal, excessive immune responses.

A technique has been reported to eliminate alveolar macrophages in mice, and these mice exhibit a significantly increased adaptive response to intra-tracheally administered antigen, compared to sham-treated controls [53]. The techniques that were used by Thepen et al. [53] to eliminate and detect alveolar macrophages could arguably kill and detect dendritic cells exposed to the alveolar epithelial surface. The excessive immune response found in the mice could still be considered comparable to the results presented in Figure 3c.

Transgenic mice have been created that can be induced to have their macrophages eliminated, but in these mice dendritic cells are affected as well [54]. After macrophage elimination the mice exhibit some of the same anatomical abnormalities described above for the RelB-/- mice such as splenomegaly, they also have enlarged lymph nodes and have impaired ability to fight infection [45-47].

### Simulation experiments with more of certain agent Types added at immune activation

**Figure 4:**
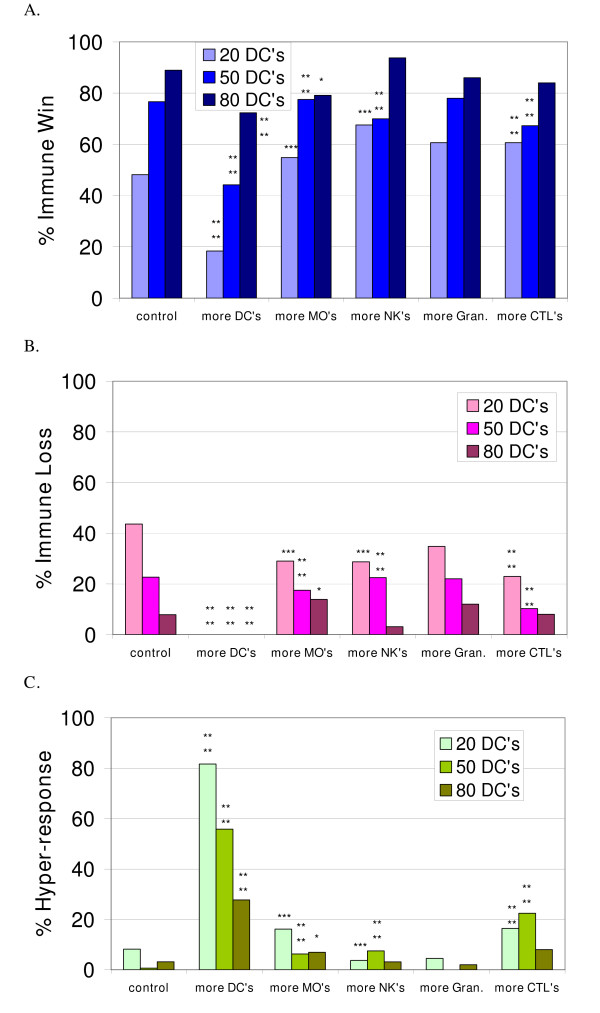
**The effect of adding more agents to the simulated immune response at activation**. Figures 4a, 4b and 4c show the percent of simulation runs that ended with the *immune win, loss and hyper-response *outcomes, respectively, when more of the indicated agent type was recruited, in combination with initial conditions of 20, 50 or 80 DCs. The control in each case is the same as shown in Figures 2 and 3. More DCs added with 20 DCs, n = 49; with 50 DCs, n = 52; with 80 DCs, n = 83; more CTLs added with 20 DCs, n = 61; with 50 DCs, n = 107; with 80 DCs, n = 50; more MΦs added with 20 DCs, n = 62; with 50 DCs, n = 80; with 80 DCs, n = 72; more NKs added with 20 DCs, n = 108; with 50 DCs, n = 80; with 80 DCs, n = 96; more Gran added with 20 DCs, n = 89; with 50 DCs, n = 50; with 80 DCs, n = 50. The asterisks indicate significant differences from the control conditions using the Chi-squared test. The p-values are as follows: **** p <= 0.0001, *** p <= 0.0015, ** p <= 0.005, * p <= 0.01.

Next, more of the innate agent types and the CTLs were added at the time of immune activation to determine the effect (Figure 4). The new values were: NumDCToSend = 2, NumMoToSend = 10, NumNKToSend = 8, and NumCTLToSend = 3 (vs. default values of 1, 5, 4, and 1, respectively, in Additional file 17). The numbers of CTL agents were increased because they did not participate in the *immune hyper-response *in the experimental results shown in Figures 2 and 3.

In Figure 4 the statistically significant differences from control are marked by asterisks and the results were analyzed in the same manner as described for Figure 3. The increased proliferation rate of CTLs (addition of more CTLs upon activation) was not beneficial but caused the *immune hyper-response *due to excessive proliferation of CTLs to occur (Table 3). Interesting results were observed when more DCs were recruited after DC activation. The simulated recruitment of more DCs to a tissue after a pathological challenge has been detected had a marked detrimental effect (more *immune hyper-response*), as opposed to having more DCs (from about 50 to 80 for these experimental conditions) present for tissue surveillance before a pathological challenge took place. This is akin to the pathology seen in psoriasis and the latter phase of atopic dermatitis [41,44]. In contrast, increasing the number of NKs recruited was significantly beneficial in the 20 DCs initial condition. More NKs aid in rapidly eliminating infected PCs.

### Simulation output data for quantities of activated agents in zone 2

**Figure 5:**
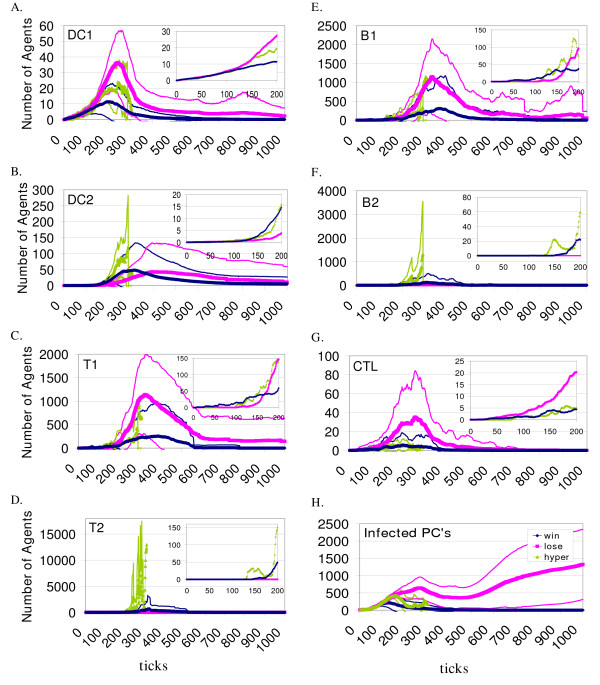
**Quantities of activated adaptive immune agents participating in the simulated immune response**. The numbers of agents participating in the viral infection simulation (109 runs with the 20 DCs starting conditions) for selected agent types are shown. The data are grouped by the outcome of each simulation run. Blue diamonds represent the mean of the immune wins (n = 58), pink squares represent the mean of the immune losses (n = 48) and green triangles represent the mean of the immune hyper-response data (n = 9), for every tick of the simulation runs (see inset in Figure 5h). The fine lines of matching color represent the standard deviation for each outcome at every tick. The inset plots contain the same data means (as the plots that contain them) for the initial ticks of the simulation, on a scale to show greater detail. Except for part h which shows data from infected Parenchymal agent counts in Zone 1, all of the other agent counts were recorded from Zone 2. Note that the scales for the numbers of agents differ for each plot.

To further explore the agent behavior that leads to different outcomes with the same initial conditions we examined the recorded output from the simulation runs. Representative output values with the starting conditions of 20 DCs are shown in Figure 5. These data are from the same simulation runs included in Figures 2, 3 and 4 for the 20 DCs starting condition. The 20 DCs initial condition was used because runs with the *immune hyper-response *and *immune loss *outcome were available to average. The continuous counts of these activated agents were selected because they were involved in the activity that was necessary for the contact-mediated information exchange that occurs in Zone 2, the lymphoid tissue zone. In parts a through g of Figure 5 the average quantities of the indicated agent types that were present in Zone 2 are plotted for every tick of the simulation. Note that only agents in the activated state are included in the figure, more agents were present that were not in the activated state. Figure 5h shows the number of infected PCs that were present in Zone 1. This reflects the course of the infection, with disappearance of infected PCs in the *immune win *outcome. In most cases, the infected PC agents were eliminated in the *immune hyper-response *outcomes, but data are only available for approximately 300 ticks because these runs were terminated early. The DCs found and activated T1s earlier when the *immune wins *occurred than in the runs when the *immune losses *occurred for the 20 DCs starting condition shown in Figure 5c (p < 0.0001, Wilcoxon Rank Sums test) and in the 50 DCs starting condition (p = 0.0016, Wilcoxon Rank Sums test; not shown). This is expected behavior because the dendritic cell-T cell interaction is necessary to mount the adaptive response.

**Figure 6:**
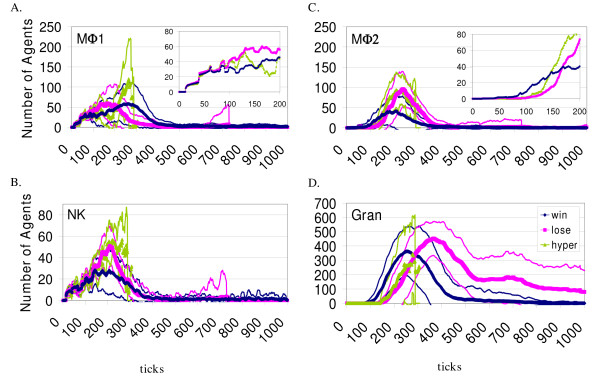
**Quantities of agents representing innate immune components participating in the simulated response**. For the same 109 simulation runs shown in Figure 5, the numbers of agents participating were recorded for Zone 1 and the data for selected agent types are shown. Only activated agents are included. The data are grouped by outcome and color coded as in Figure 5.

Data derived from the participation of the agents representing the cells of the innate immune system in Zone 1 is shown in Figure 6. Recruitment of pro-inflammatory MΦ1s precedes the recruitment of anti-inflammatory MΦ2s, as expected (they enter in a naïve state). MΦ1 presence peaks later and persists for a longer duration in the *immune win *outcome than the *immune loss *outcome. MΦ2s persist longer in the *immune loss *outcome. The same may be said for the recruitment of Granulocyte agents, more of them are present and for a longer duration in the *immune loss *outcome.

In general, *immune wins *involved the efficient participation of the necessary agent types in the simulated immune response, with fewer activated agent numbers recorded compared to the *immune losses *(Figures 5 and 6). The simulation runs classified as *immune losses *involved the delayed participation of much greater numbers of agents, because the spreading viral infection provided a greater stimulus to recruitment and proliferation. Figure 5h shows that on average, far more infected cells are present in the *immune loss *outcome, and the least are present in the *immune win *outcome. Enlarged, hypertrophic lymph nodes are a common clinical finding in the face of extensive infection, and we believe that the *immune loss *outcome pattern in Zone 2 reflects this phenomenon. The tissue damage (more dead PCs, data not shown) and extensive Granulocyte agent and MΦ participation seen in this outcome (Figure 6) is clinically relevant as well [55].

The stochastic aspect of the simulator can be appreciated from the results presented in Figures 5 and 6. The variability is shown in the standard deviation plotted for every tick. This is consistent with the observed stochasticity seen in the regulation of the immune response [56], as well as in the obvious experience of whole-animal experimental preparations and in the clinical setting.

### Mechanisms found to produce the immune loss outcome

If too few DCs, NKs or MΦs are initially present in Zone 1, it is more likely that an infection will progress further before it is recognized by these innate immune components. NKs and MΦs will "kill" infected PCs when they detect them, having the potential to eliminate infected PCs without adaptive immune response involvement. When these agent numbers are deficient the stimulus for activation will be greater when it is finally recognized, and more DCs will be recruited and sent to Zone 2. This is the situation in the *immune loss *outcome as well as the *immune hyper-response*. In both cases, more activated cells are generated to fight the infection.

### Mechanisms found to produce the immune hyper-response

The initial experimental conditions leading to more frequent hyper-response outcomes suggest potential mechanisms for hypersensitivity reactions. Insufficient numbers of DCs in combination with insufficient numbers of NKs and MΦs, insufficient numbers of NKs alone, and increased DC recruitment after activation are the conditions most likely to produce this outcome. The positive feedback behavior that has been observed in the simulation begins with the DC presenting antigen to the T-cell agents specific for the antigen in Zone 2. T-cell agents proliferate, increasing the likelihood of contact with a DC presenting antigen, thus leading to further proliferation in Zone 2. Both DCs and Ts activate antigen-specific Bs, so Bs can be seen to be proliferating excessively as well (Table 3). Apoptosis of the DCs can put an end to this loop, by removing the stimulus for proliferation. Experiments to test this hypothesis are described in the next section.

### Results from experiments with DCs that are unable to undergo apoptosis

The role for apoptosis of dendritic cells in controlling T cell-mediated immune responses in the skin [57], lungs [58], gut [59], and systemically [60] has been examined. Matsue et al. [57] showed that after presenting antigen to T cells in secondary lymphoid tissue, dendritic cells died via apoptosis. Mice with dendritic cells that lacked CD95 (Fas, a receptor needed for apoptosis), had enhanced ability to cause delayed-type hypersensitivity when their antigen primed dendritic cells were injected into the footpads of naïve mice that were then challenged with antigen. They concluded that dendritic cell apoptosis is an important mechanism for controlling T cell activation.

Julia et al. [58] identified unusual dendritic cells that persisted for excessive periods of time in the lungs of mice in a murine model of asthma. These were mature, antigen presenting dendritic cells that maintained the presence of antigen-specific T_HELPER_-2 cells in the lung. In a murine model of cow's milk allergy, Man et al. [59] observed that mice with the allergy had dendritic cells that were resistant to T-cell mediated apoptosis compared to non-allergic mice.

Chen et al. [60] have reported that disruption of the mechanism for apoptosis specifically in the dendritic cells of mice leads to enhanced capacity to induce antigen specific immune responses measured as (CD4+ and CD8+) T lymphocyte proliferation, chronic increased lymphocyte activation without known antigen stimulus, and increases in the incidence of autoimmune pathology. To demonstrate the similarity of the *immune hyper-response *outcome in the BIS, simulation experiments were performed with the apoptosis mechanisms programmed into DCs effectively turned off [see Additional files 3 and 4]. The input parameters LIFE_DC_Zone1 and LIFE_DC_Zone2 were set to 1000 ticks, and LIMIT_NUM_Ts was set to 10000 to prevent apoptosis of DCs from T contacts [see Additional file 17]. The result was that all of the 66 simulation runs with the 50 DCs starting condition ended in the *immune hyper-response*. The agent types involved are listed in Table 3. In many of the runs, the numbers of DCs present at termination also exceeded 1000 (data not shown). The results from the simulation are similar to the pathological conditions observed in the mice. These results are also comparable to the experimental results shown in Figure 4 with an increase in the *immune hyper-response *due to more DCs entering the tissue after immune activation has taken place.

### Hypersensitivity reactions to viral infection in vivo

While hypersensitivity reactions generally involve non-infectious environmental elements, there are examples of viruses that cause harmful immune responses, such as the Respiratory Syncytial Virus (RSV) [61]. The damage caused by the immune system in this disease is mediated by downstream effects of the T_HELPER_-2 lymphocyte participation. A predisposition to asthma and allergy is associated with early RSV infection [62]. Rhinoviruses have also been implicated in the etiology of asthma (reviewed in [63]). The persistence of these viruses in children with insufficient innate immune responses has been found to correlate with hypersensitive pulmonary disease. The incidence of hypersensitivity disorders is approximately 10–15% of the western population and rising in the developed world [64]. The correlation between the hypersensitivity reactions of the immune system that involve T lymphocytes and dendritic cells and the emergent *immune hyper-response *is a striking example of a matching behavioral pattern found with the BIS. The prevalence of the diseases involving immune disregulation makes a computer simulation to study the disease mechanisms a valuable tool.

The data output from the BIS also allows the examination of conditions leading to successful or unsuccessful elimination of virally infected PCs. The events that must take place for the adaptive immune response to be initiated occur in the lymph nodes, reflected in behavior seen in Zone 2. Continuous, comprehensive cell (and cytokine) quantification in the lymph node or spleen for an individual's immune response to a specific pathogen is not possible in a living system. Only in recent years has two-photon microscopy allowed three-dimensional imaging of live lymphoid tissue (with fluorescently labeled cells), providing a means for estimation of the rate of dendritic cell-T cell contacts that must occur in lymph nodes for initiation of the adaptive response [30]. Traditionally, time course data has come from *in vitro *experiments or from *in vivo *studies with "snap-shots" of one time point per animal, because the animals must be sacrificed to get the data. In this way the data shown in Figures 5 and 6 is unique, and any comparison with time course data in the literature should be made with this in mind.

## Conclusion

One of the greatest challenges facing the biomedical research community today is the issue of biocomplexity. The advance of science in the modern age has been predicated upon the paradigm of linear reductionism, i.e. reducing a system into a series of linear relationships that can then be subjected to experimental analyses, and subsequent reconstruction of the system from the results of those experiments. Reductionism has been so successful because it is the only way to obtain an approximation of cause and effect and thereby gain insight into mechanisms of action. However, the recognition of the prevalence of complex, nonlinear systems in nature has lead to an acceptance in many avenues of science of the limitations of linear reductionism. The biomedical research community is one of those groups coming to grips with this challenge. What is needed, then, is a means of accomplishing "nonlinear reductionism", or a means of effectively synthesizing the information acquired from the traditional reductionist paradigm into a framework that effectively reconstructs the effects of the interactions between the various components of the system.

Towards this end, there has been great growth in the fields of "in-silico" biology. The fields of mathematical, computational and translational systems biology have all evolved to address this need for a synthetic method. To this growing area we offer the Basic Immune Simulator as a demonstration, educational and research aid for dealing with the biocomplexity of the interactions between the innate and adaptive immune responses. We believe that the agent-based structure of the BIS facilitates its translational role, providing a more intuitive approach to modeling biology. Furthermore, the rule-based emphasis of the BIS lends itself to the transparency with respect to its agent rules that is necessary for any simulation tool. Despite its abstraction, certain essential dynamics of the relationship between the innate and adaptive immune response become clear when using the BIS. Furthermore, its reliance upon the open-source paradigm allows the BIS to potentially serve as a departure point for more detailed and sophisticated models. We hope that the BIS will serve to improve the access of simulation tools to the general biomedical research community, and be additional evidence of the utility of the agent-based modeling methodology.

## Availability and requirements

A down-loadable version of the Basic Immune Simulator [24] can be found at: , and at [25]. Detailed instructions for downloading are available at the Digital Union website listed above, but they will be summarized here. The files needed to run the simulation include the BasicImmuneSimulator.jar file, the Repast J launcher  and the Java Runtime Environment (version 1.4.2 or higher, see Java SE, Java Runtime Environment [JRE]6 or Java SE Development Kit [JDK]6, at the Sun Developer Network website) [65] if one is using a PC. If one is using a Macintosh computer, one only needs to download the OS X version of Repast J [25] along with the BasicImmuneSimulator.jar file. The Repast website has detailed instructions and documentation for the Repast GUI. No programming experience is necessary to run the BIS, but Java programming skill is necessary to modify it. No license or restrictions apply to the software listed above.

## Abbreviations

ABM – Agent-based modeling, Ab1 – antibody-1, Ab2 – antibody-2, Ag – antigen, B (B1, B2) – B Cell agent (type 1 or 2), BIS – Basic Immune Simulator, C' – complement, CK1 – cytokine-1, CK2 – cytokine-2, CTL – Cytotoxic T Lymphocyte agent, DC (DC1, DC2) – Dendritic Cell agent, Gran – Granulocyte agent, GUI – graphical user interface, MΦ (MΦ1, MΦ2) – Macrophage agent (type 1 or 2), MK1 – monokine-1, MK2 – monokine-2, NA – not applicable, PC – Parenchymal Cell agent, PK1 – parenchymalkine-1, Portal – Portal agent, T (T1, T2) – T Cell agent (type 1 or 2). Underlined terms are input parameters, italicized terms are nomenclature specific to the BIS. The words "agent" and "signal" refer to elements of the BIS, and the words "cell" and "cytokine" or "chemokine" refer to living systems.

## Competing interests

The author(s) declare that they have no competing interests.

## Authors' contributions

CGO conceived of the Basic Immune Simulator and wrote the rules for the behavior of all of the agents that were present in the initial version. VAF wrote the program for the simulation, conducted the experiments, analyzed the data and drafted the initial version of the manuscript. GCA drafted portions of the manuscript and revised it critically for important intellectual content. The living authors, VAF and GCA, read and approved the final manuscript.

## Supplementary Material

Additional file 1 State diagram key. A key to the symbols used in all of the state diagrams. Click here for file

Additional file 2 Parenchymal Cell Agents (PCs) in Zone 1. A state diagram of the potential PC behavioral sequences in Zone 1. Click here for file

Additional file 3 Dendritic Cell agents (DCs) in Zone 1. A state diagram of the potential DC behavioral sequences in Zone 1. Click here for file

Additional file 4 Dendritic Cell agents (DCs) in Zone 2. A state diagram of the potential DC behavioral sequences in Zone 2. Click here for file

Additional file 5 Macrophage agents (MΦs) in Zone 1. A state diagram of the potential MΦ behavioral sequences in Zone 1. Click here for file

Additional file 6 Natural Killer Cell agents (NKs) in Zone 1. A state diagram of the potential NK behavioral sequences in Zone 1. Click here for file

Additional file 7 B Cell agents (Bs) in Zone 2. A state diagram of the potential B behavioral sequences in Zone 2. Click here for file

Additional file 8 B Cell agents (Bs) in Zone 3. A state diagram of the potential B behavioral sequences in Zone 3. Click here for file

Additional file 9 B Cell agents (Bs) in Zone 1. A state diagram of the potential B behavioral sequences in Zone 1. Click here for file

Additional file 10 T Cell agents (Ts) in Zone 2. A state diagram of the potential T behavioral sequences in Zone 2. Click here for file

Additional file 11 T Cell agents (T1s) in Zone 1. A state diagram of the potential T1 behavioral sequences in Zone 1. Click here for file

Additional file 12 T Cell agents (T2s) in Zone 1. A state diagram of the potential T2 behavioral sequences in Zone 1. Click here for file

Additional file 13 Cytotoxic T Lymphocyte agents (CTLs) in Zone 2. A state diagram of the potential CTL behavioral sequences in Zone 2. Click here for file

Additional file 14 Cytotoxic T Lymphocyte agents (CTLs) in Zone 1. A state diagram of the potential CTL behavioral sequences in Zone 1. Click here for file

Additional file 15 Granulocyte agents in Zones 1 and 3. A state diagram of the potential Granulocyte agent behavioral sequences in Zones 1 and 3. Click here for file

Additional file 16 Portal agents in Zones 1, 2 and 3. A state diagram of the potential Portal agent behaviors in Zones 1, 2 and 3. Click here for file

Additional file 17 Input parameters for simulation runs. A table of all of the input parameters and the Zones that they affect. Click here for file
