# Supplementary material for: The Basic Immune Simulator: An agent-based model to study the interactions between innate and adaptive immunity
Source: Theor Biol Med Model. 2007 Sep 27;4:39. doi: 10.1186/1742-4682-4-39 (PMC2186321; doi:10.1186/1742-4682-4-39)
Supplement: Additional file 1 — State diagram key. A key to the symbols used in all of the state diagrams. [file 1742-4682-4-39-S1.pdf]

Symbol	Meaning
State 0	The initial state
Blue text	A description of the state
ALL CAPS	A signal is produced by the agent in the state
Red text	A condition that causes a transition to another state
ALL CAPS	A signal that causes a state transition
Magenta text	A state transition that involves addition of agents
Some input value	An input parameter value
Initialization	Conditions existing at the beginning of a simulation run
·········›	A state transition, usually annotated with what causes it
Zone X	An arrow to/from a box like this indicates migration to/from another Zone
State X	A final state, death

Additional file 1: State Diagram Key.

All of the state diagrams for the behavior of the agents use the same symbols. Any input parameters referred to from Additional file 17 are underlined.
