# Supplementary material for: The Basic Immune Simulator: An agent-based model to study the interactions between innate and adaptive immunity
Source: Theor Biol Med Model. 2007 Sep 27;4:39. doi: 10.1186/1742-4682-4-39 (PMC2186321; doi:10.1186/1742-4682-4-39)
Supplement: Additional file 2 — Parenchymal Cell Agents (PCs) in Zone 1. A state diagram of the potential PC behavioral sequences in Zone 1. [file 1742-4682-4-39-S2.pdf]

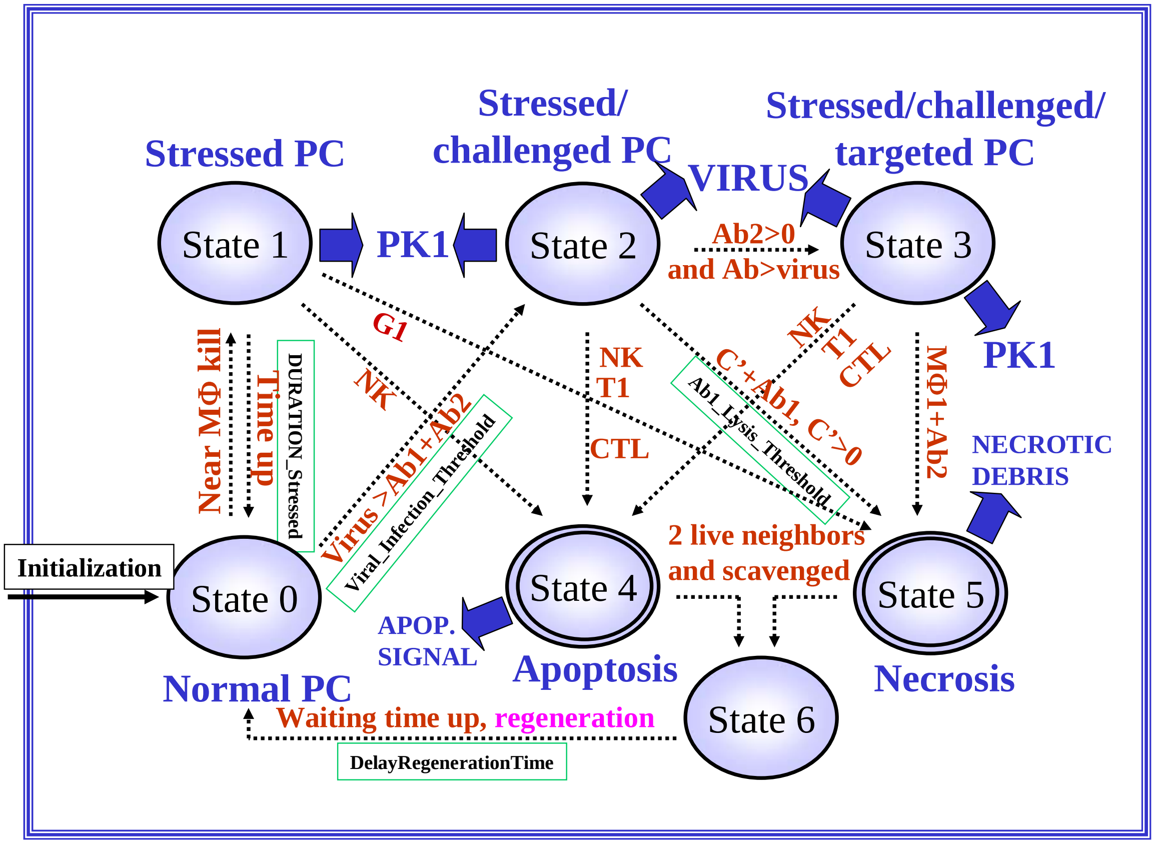

Additional file 2. State Diagram: Parenchymal Cell Agents (PCs) in Zone 1.

PCs remain stationary in Zone 1 for the duration of the simulation. They begin as healthy, functional agents (State 0) that become infected with a virus and emit virus signal and Parenchymal-kine 1 (PK1), the stress signal (States 2 and 3). PK1 represents mediators such as heat-shock proteins [66], uric acid [67] or chemerin [68] that may be emitted by cells undergoing stress. PCs have two boolean properties or variables that reflect their well-being. One is the property of being stressed, which is true in States 1-3. The other is the property of being challenged, which for the experiments described herein is infection with a virus (true in States 2 and 3). The "challenge" for a simulation run is an input parameter (Additional file 17, Set_ViralInfection) that drives the immune response. Challenged PCs are always stressed, but PCs may be stressed without being challenged (State 1). Virally infected PCs may then transition to one of three fates. They may be killed by Natural Killer agents (NKs), pro-inflammatory T Cell agents (T1s) or Cytotoxic T Lymphocyte agents (CTLs) and undergo apoptosis (State 4). They may be bound by antibody (Ab; State 3) making them a target for recognition by pro-inflammatory Macrophage agents (MΦ1s) [73]. Or, they may be lysed by complement products (C') if Antibody 1 (Ab1) is present [70]. Both of the latter lead to death with release of necrotic debris (State 5). A PC that is in the immediate vicinity of another PC being killed by a MΦ1 will become stressed, representing damage due to reactive oxygen species release by the MΦ1 (State 1) [55]. Such a stressed PC then releases PK1 and may be killed by an NK [72]. PCs stressed for any reason may also be killed by exposure to Degranulation product 1 (G1), the degranulation signal released by Granulocyte agents [55, 74]. This results in the release of the signal for necrotic debris (State 5).

The dead PC debris must be scavenged by a MΦ before any regeneration of the PC may occur (State 6) [75]. A healthy PC replaces a PC that is dead after the dead PC has been scavenged and if there are at least two healthy PCs in the spaces proximal to the space of the dead PC. This represents the division of one of the healthy PCs. There is a short waiting period once these conditions are met (DelayRegenerationTime) and then the new PC replaces the dead one (State 0).
