# Supplementary material for: The Basic Immune Simulator: An agent-based model to study the interactions between innate and adaptive immunity
Source: Theor Biol Med Model. 2007 Sep 27;4:39. doi: 10.1186/1742-4682-4-39 (PMC2186321; doi:10.1186/1742-4682-4-39)
Supplement: Additional file 3 — Dendritic Cell agents (DCs) in Zone 1. A state diagram of the potential DC behavioral sequences in Zone 1. [file 1742-4682-4-39-S3.pdf]

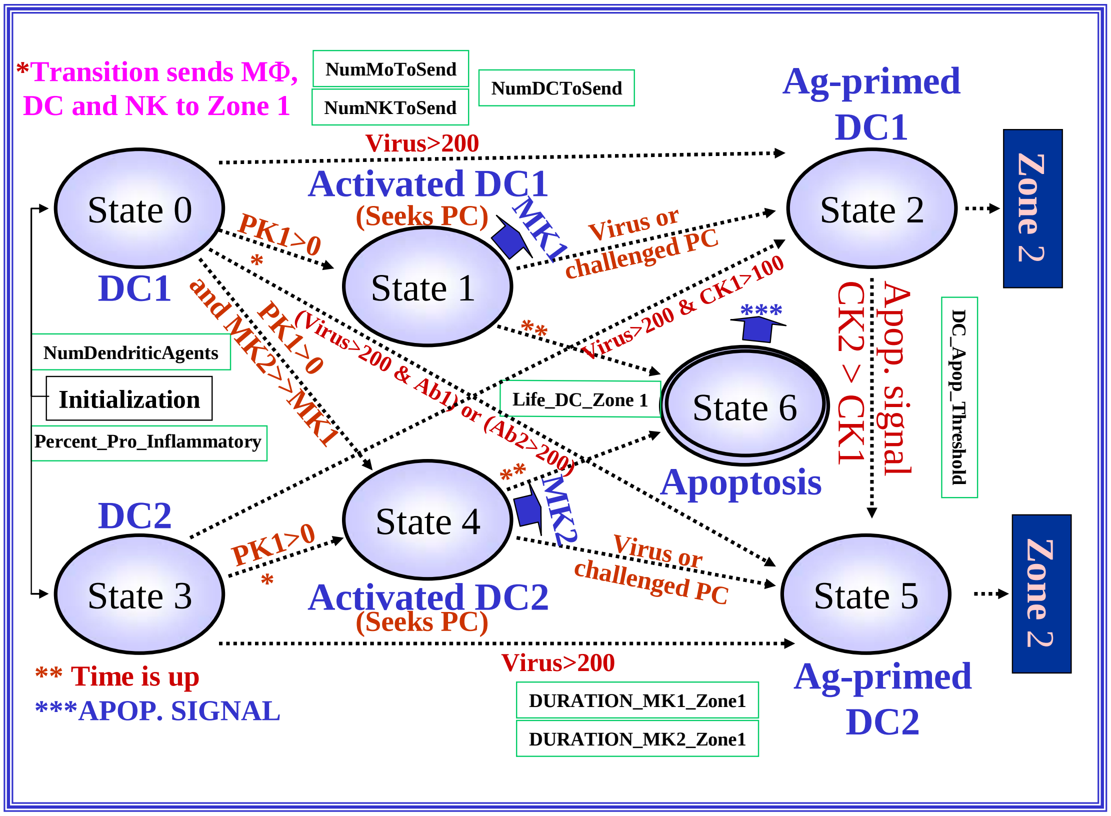

Additional file 3. State Diagram: Dendritic Cell Agents (DCs) in Zone 1.

DCs begin in Zone 1, where they function in surveillance of the tissue for any disruption of the healthy state of the tissue [38]. At initialization they may be in State 0 or State 3, depending on whether they have the potential to promote inflammation (DC1) or down-regulate it (DC2). The number of DCs and the ratio of DC1:DC2 is controlled by the input parameters NumDendriticAgents and Percent_Pro_Inflammatory (Additional file 17). These subsets are meant to represent the ability of dendritic cells to polarize the immune response [80, 81, 86]. The numbers of DCs initially in Zone 1 used in the experiments approximate what may be found in normal dermis (% of cells) [33]. When the simulation begins, the DCs migrate randomly in Zone 1, able to detect soluble stress factor (PK1), virus, antibodies (Ab1 or Ab2), and pro- and anti-inflammatory cytokines [Mono-kine 1 (MK1) and Mono-kine 2 (MK2), Cytokine 1 (CK1) and Cytokine 2 (CK2); see Table 1]. The DCs may transition to another state depending on which signal they encounter first. PK1 causes the DCs to become activated [76] and transition to States 1 or 4. At this time NKs, MΦs and a DC enter Zone 1, as they would in response to chemokines. The numbers that enter are controlled by the input parameters NumNKToSend, NumMOToSend and NumDCToSend. Once the DCs detect PK1 they follow its concentration gradient, seeking a challenged PC. In the activated state DC1s and DC2s also release signal, MK1 or MK2 (respectively). At this point a DC1 may be induced to convert to the down-regulatory DC2 phenotype by the preponderance of MK2 signal already in the environment [77]. In addition to the transitions to the activated states, the presence of virus in the immediate environment causes the transition to the antigen-primed state (State 2 or 5) [79]. Viral antigen bound by antibody induces the transition of a DC1 to the DC2 type [94] and virus in combination with pro-inflammatory CK1 causes the transition of a DC2 to the pro-inflammatory, antigen-primed DC1 state [78]. Contact with virally infected PCs also causes activated DC1s and DC2s to transition to their respective antigen-primed states [38].

If DC1s or DC2s reach the activated states (States 1 or 4) but do not detect soluble (signal) or PC-bound antigen within a pre-defined number of ticks they undergo apoptosis, or programmed cell death (State 6; LIFE_DC_Zone1) [90]. Once the DCs do make contact with antigen, they migrate to Zone 2 to present the antigen to the B Cell agents (Bs), Ts and Cytotoxic T Lymphocyte agents (CTLs) [38]. If the DC1 senses a preponderance of CK2 or detects apoptotic debris at the tick when the transition to Zone 2 is to be made, it will convert to a DC2 before migrating [76, 77].
