# Supplementary material for: The Basic Immune Simulator: An agent-based model to study the interactions between innate and adaptive immunity
Source: Theor Biol Med Model. 2007 Sep 27;4:39. doi: 10.1186/1742-4682-4-39 (PMC2186321; doi:10.1186/1742-4682-4-39)
Supplement: Additional file 4 — Dendritic Cell agents (DCs) in Zone 2. A state diagram of the potential DC behavioral sequences in Zone 2. [file 1742-4682-4-39-S4.pdf]

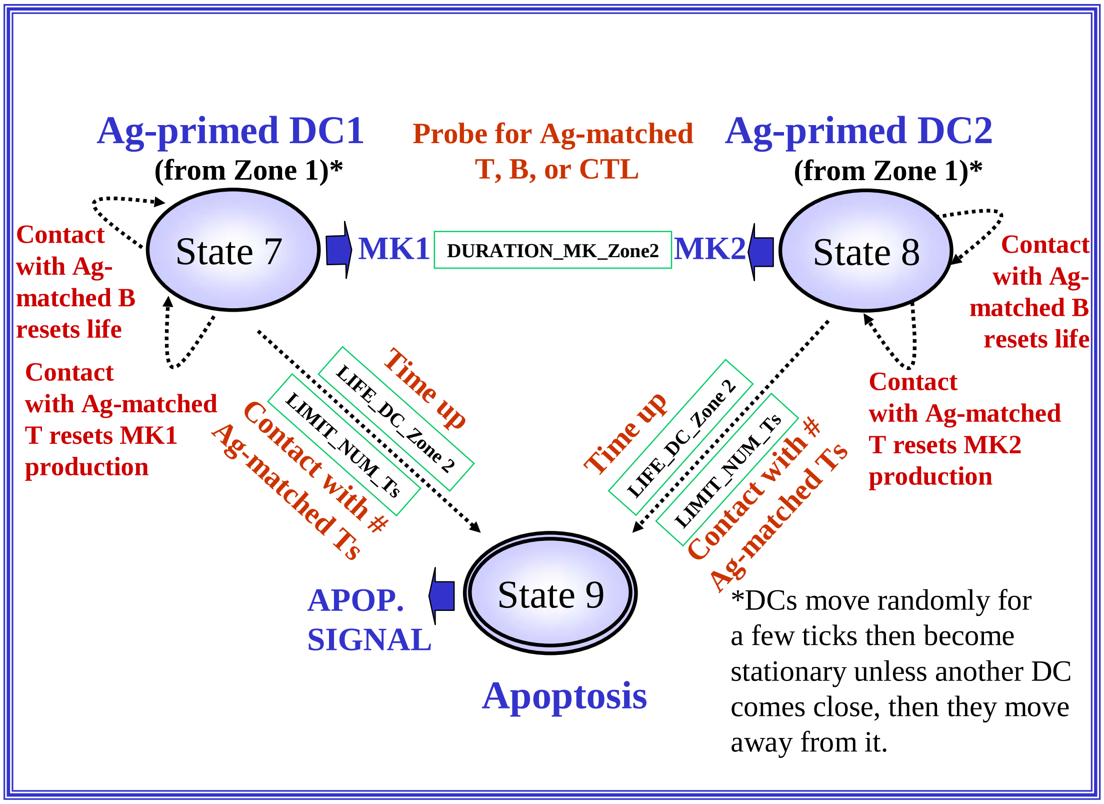

Additional file 4. State Diagram: Dendritic Cell Agents (DCs) in Zone 2.
Once the DCs have migrated to Zone 2, they move randomly for a set number of ticks and then become stationary. They thereafter move only to maintain an evenly distributed web of DCs in Zone 2 [88]. While in Zone 2 they produce their respective cytokines and probe all of the B Cells Agents (Bs), T Cell Agents (Ts) and Cytotoxic T Lymphocytes (CTLs) that move randomly within their reach to look for an antigen-specificity matched agent. All of the agents in Zone 2 have a pre-set specificity, and the number of ticks that will elapse before a matching agent is detected by a DC is random. When a match is found, the B, T or CTL becomes cognizant of the contact, affecting its state. The contact with an antigen-matched agent is noted by the DC, and it may extend the life of the DC [91], restart signal production or lead to the apoptosis of the DC if the number of T contacts reaches a threshold amount (LIMIT_NUM_Ts) [83, 89].
