# Supplementary figures and images for: The Basic Immune Simulator: An agent-based model to study the interactions between innate and adaptive immunity
Source: Theor Biol Med Model. 2007 Sep 27;4:39. doi: 10.1186/1742-4682-4-39 (PMC2186321; doi:10.1186/1742-4682-4-39)

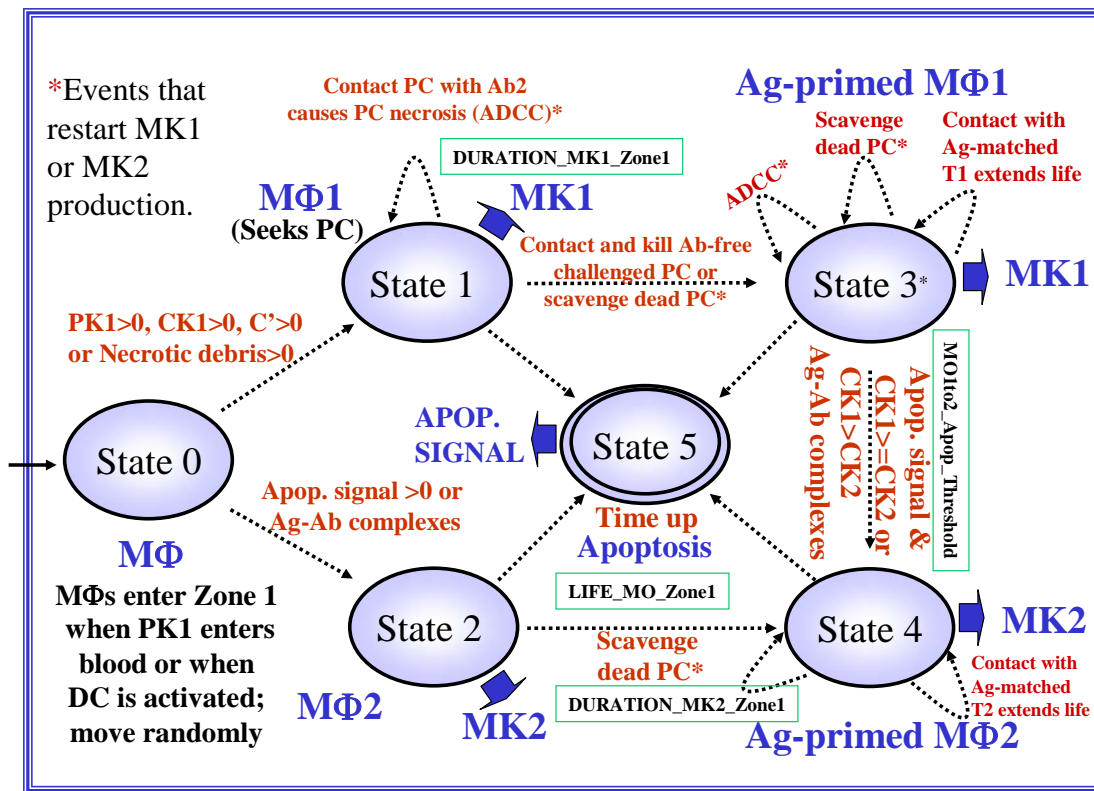

Supplement: Additional file 5 — Macrophage agents (MΦs) in Zone 1. A state diagram of the potential MΦ behavioral sequences in Zone 1. [file 1742-4682-4-39-S5.pdf]

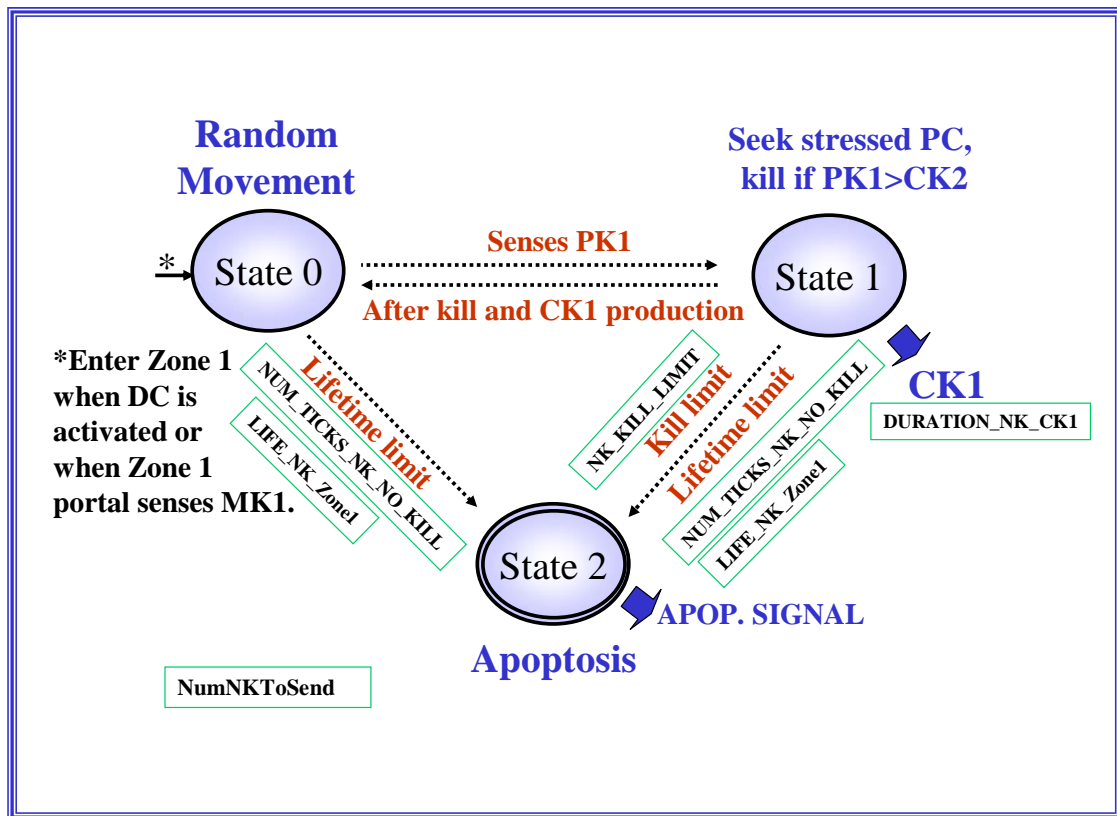

Supplement: Additional file 6 — Natural Killer Cell agents (NKs) in Zone 1. A state diagram of the potential NK behavioral sequences in Zone 1. [file 1742-4682-4-39-S6.pdf]
