# Supplementary material for: The Basic Immune Simulator: An agent-based model to study the interactions between innate and adaptive immunity
Source: Theor Biol Med Model. 2007 Sep 27;4:39. doi: 10.1186/1742-4682-4-39 (PMC2186321; doi:10.1186/1742-4682-4-39)
Supplement: Additional file 7 — B Cell agents (Bs) in Zone 2. A state diagram of the potential B behavioral sequences in Zone 2. [file 1742-4682-4-39-S7.pdf]

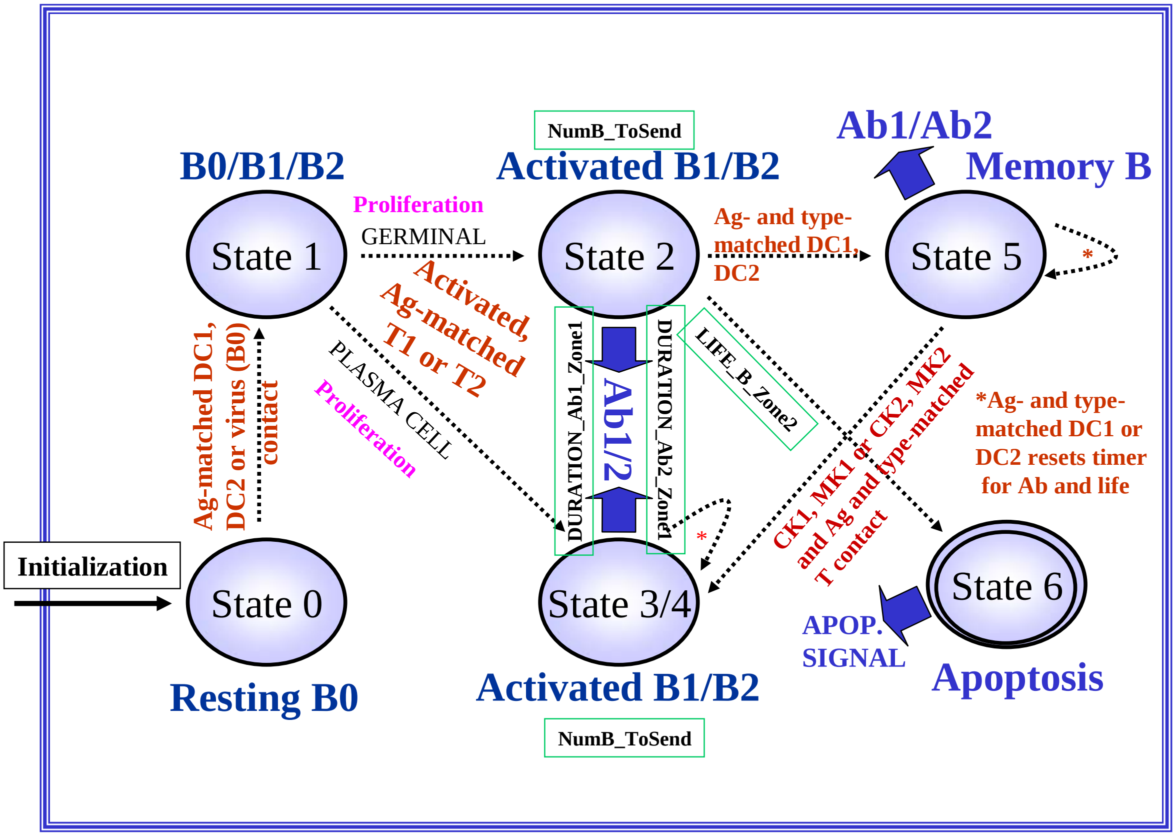

Additional file 7. State Diagram: B Cell agents (Bs) in Zone 2.

Bs begin in a resting state in Zone 2. They require contact with antigen (Ag) or a Dendritic Cell agent (DC) that is presenting antigen that matches their pre-set specificity [82]. The fraction of Bs that is specific for any particular antigen is an input parameter to the simulation (<u>PercentBAntiViral</u>). The default fraction of specific Bs that was used for these experiments was 2% (Appendix B).

The DC also controls the response type of the B. If presentation of antigen is made by a DC1, the B will be type B1 and make antibody-1 (Ab1). Presentation by a DC2 will cause the B to become a B2 and make Ab2 (Table 1). The abbreviations B1 and B2 indicate different populations present in the simulation and are not meant to correspond to such designations in the literature for B lymphocytes [105]. If the B detects antigen available freely in the lymphatic fluid, any activated T Cell agent type (T1 or T2) that subsequently contacts the B will determine its response type.

Once the B has either seen soluble Ag (such as virus) or seen Ag presented by a DC, the B requires contact with an antigen matched T1 or T2 before it can produce Ab (States 2-5) [36]. Once the B makes contact with an activated, Ag-matched T, it may take one of two (stochastically determined) paths. It may become a germinal B (State 2), producing antibody and remaining in Zone 2, or a plasma cell (States 3 or 4), producing antibody and traveling to Zone 3. Specific T contact also causes the B to proliferate, the number of progeny produced at each contact is an input parameter to the simulation (<u>NumB_ToSend</u>). A sufficient number of subsequent Ag-specific DC contacts results in the formation of a long-lived memory B (State 5), with each contact extending the life of the B. A memory B may be reactivated (to States 3 or 4) by the contact of an Ag-specific T and the presence of cytokines in Zone 2.
