# Supplementary material for: The Basic Immune Simulator: An agent-based model to study the interactions between innate and adaptive immunity
Source: Theor Biol Med Model. 2007 Sep 27;4:39. doi: 10.1186/1742-4682-4-39 (PMC2186321; doi:10.1186/1742-4682-4-39)
Supplement: Additional file 8 — B Cell agents (Bs) in Zone 3. A state diagram of the potential B behavioral sequences in Zone 3. [file 1742-4682-4-39-S8.pdf]

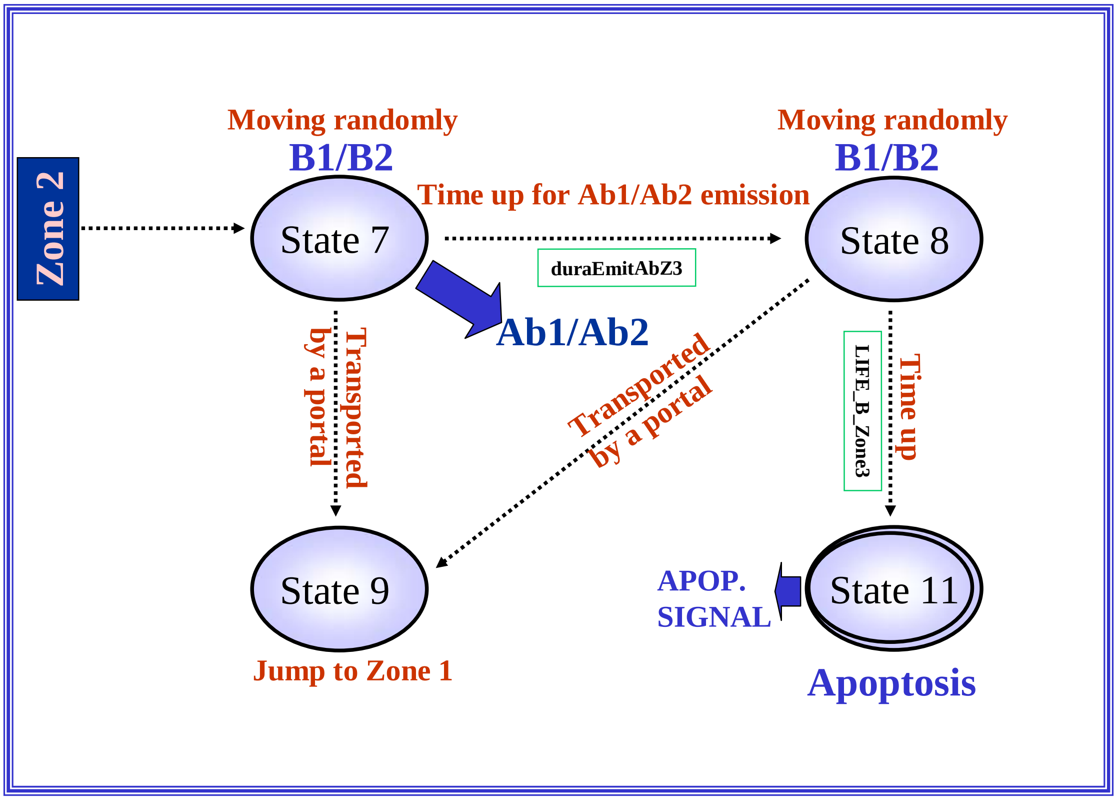

Additional file 8. State Diagram: B Cell agents (Bs) in Zone 3.
Activated Bs in States 3 or 4 represent plasma cells and migrate to Zone 3 where they produce antibody (Ab) that diffuses into Zone 1, and they may migrate into the actual site of inflammation, Zone 1, from there (State 9) [104]. As long as they remain in Zone 3 they move randomly. Bs have a finite lifetime (LIFE_B_Zone3) and a finite period of time to produce Ab in Zone 3 (duraEmitAbZ3) determined by input parameters.
