# Supplementary material for: The Basic Immune Simulator: An agent-based model to study the interactions between innate and adaptive immunity
Source: Theor Biol Med Model. 2007 Sep 27;4:39. doi: 10.1186/1742-4682-4-39 (PMC2186321; doi:10.1186/1742-4682-4-39)
Supplement: Additional file 9 — B Cell agents (Bs) in Zone 1. A state diagram of the potential B behavioral sequences in Zone 1. [file 1742-4682-4-39-S9.pdf]

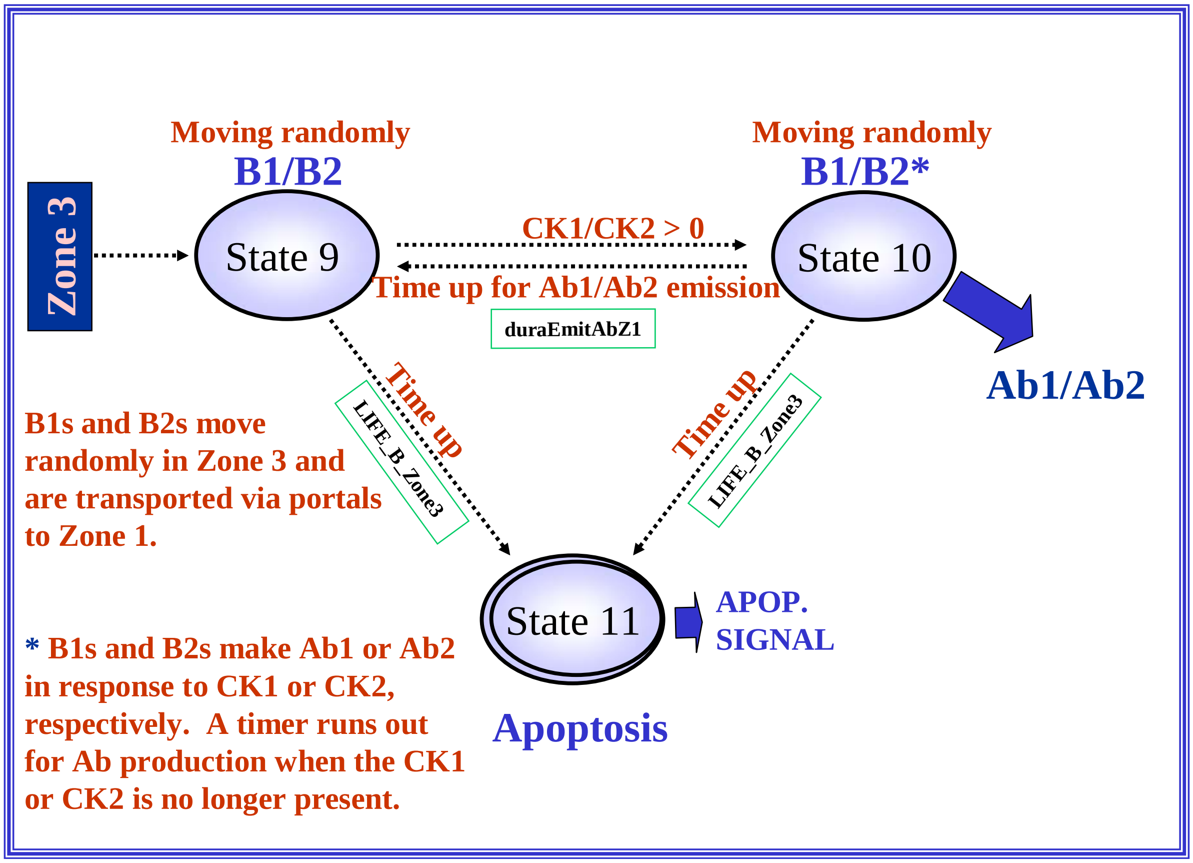

Additional file 9. State Diagram: B Cell agents (Bs) in Zone 1.
In Zone 1 the Bs continue to move randomly and produce antibody (Ab) if cytokine-1 (CK1) or CK2 is present (State 10; duraEmitAbZ1) [103]. Once the signal is no longer present they cease to produce Ab within a period of time (State 9). This represents the behavior of B lymphocytes in immune responses where they may be found in sites of tissue inflammation [104]. Bs have a finite lifetime in Zone 1 determined by an input parameter (Additional file 17; LIFE_B_Zone3).
