# Supplementary material for: The Basic Immune Simulator: An agent-based model to study the interactions between innate and adaptive immunity
Source: Theor Biol Med Model. 2007 Sep 27;4:39. doi: 10.1186/1742-4682-4-39 (PMC2186321; doi:10.1186/1742-4682-4-39)
Supplement: Additional file 10 — T Cell agents (Ts) in Zone 2. A state diagram of the potential T behavioral sequences in Zone 2. [file 1742-4682-4-39-S10.pdf]

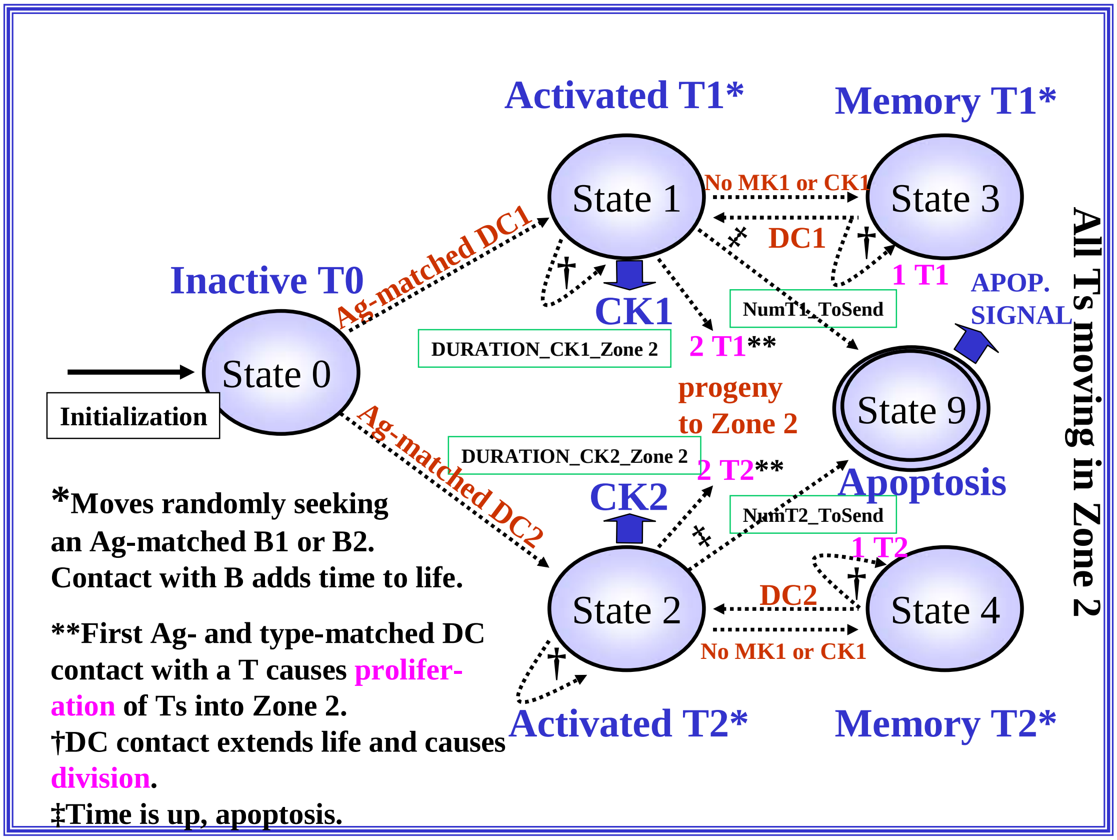

Additional file 10. State Diagram: T Cell agents (Ts) in Zone 2.

The Ts in the simulation represent lymphocytes of the T-HELPER type. Like the B Cell agents (Bs), the Ts begin in Zone 2 in an inactive state, moving randomly and waiting to make contact with an antigen-matched Dendritic Cell agent (DC). The pro-inflammatory (DC1) or down-regulatory (DC2) type of the DC that makes first contact with the T determines the type of response that the T will promote in the remainder of the simulation (T1 or T2) [38, 81, 83, 86]. The initial fraction of Ts that are specific for any given antigen is an input parameter to the simulation, and the default value is 0.4% (Additional file 17; PercentTAntiViral).

Once contact with an antigen-matched DC is made, the T is activated (States 1 or 2) and produces cytokine-1 or -2 (CK1 or CK2), depending on its response type. Proliferation of the T1 or T2 also occurs at this first contact (NumT1_ToSend or NumT2_ToSend). Subsequent contacts with antigen-matched DCs extend the life of the T [96]. The Ts also probe any Bs in proximal locations for their antigen specificity. An antigen-matched contact with a B affects the B if it has already been activated by a DC [95]. The contact also extends the life of the T. Ts in State 1 or 2 monitor the presence of CK1 and CK2 in their immediate environment. Absence of cytokines in the environment allows them to transition to long-lived memory Ts. Contact with an antigen- and response type- matched DC brings them back to the activated state [96]. In the absence of DC contact in Zone 2 activated Ts may undergo apoptosis [91]. Ts migrate to Zone 3, where they move randomly until they migrate into Zone 1.
