# Supplementary material for: The Basic Immune Simulator: An agent-based model to study the interactions between innate and adaptive immunity
Source: Theor Biol Med Model. 2007 Sep 27;4:39. doi: 10.1186/1742-4682-4-39 (PMC2186321; doi:10.1186/1742-4682-4-39)
Supplement: Additional file 11 — T Cell agents (T1s) in Zone 1. A state diagram of the potential T1 behavioral sequences in Zone 1. [file 1742-4682-4-39-S11.pdf]

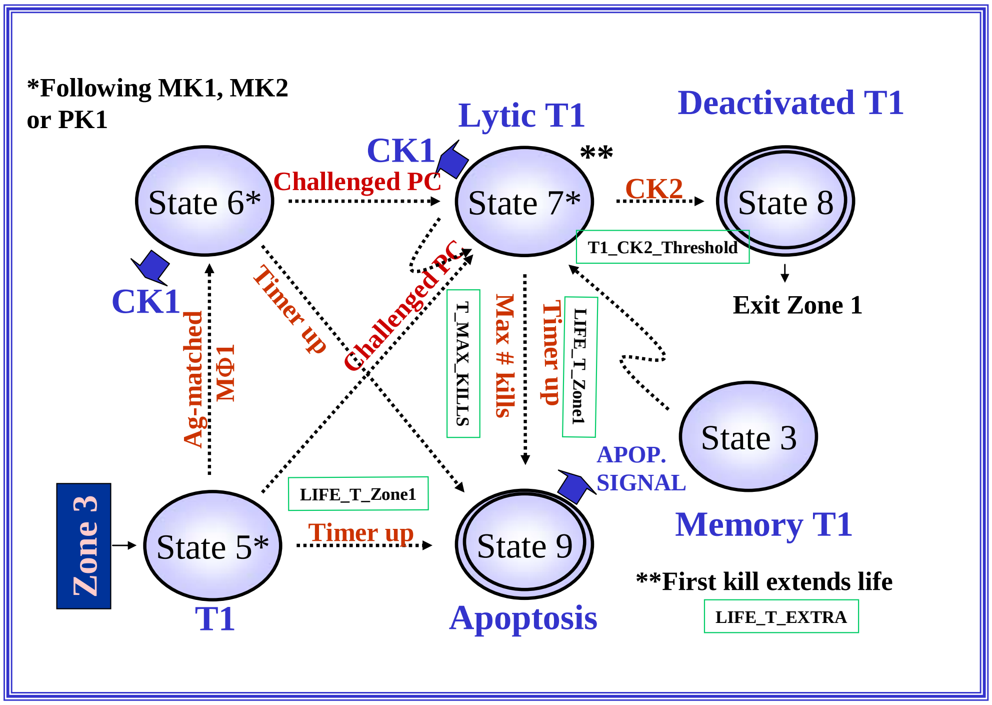

Additional file 11. State Diagram: T Cell agents (T1s) in Zone 1.

When T1s arrive in Zone 1 they may follow monokine-1 (MK1), MK2 or parenchymalkine-1 (PK1), whichever signal they find to be the strongest in their immediate vicinity. If none are present they move randomly until they encounter a signal. They probe their immediate environment for the presence of an antigen-matched Macrophage agent 1 (MΦ1), and if they encounter one they begin to emit cytokine-1 (CK1) [85]. They also probe their immediate environment for a virally infected Parenchymal Cell agent (PC), which they kill upon encounter. The T1s count the number of PCs that they kill, because they may only kill a finite number of times before undergoing apoptosis themselves (T_MAX_KILLS) [71]. If they detect CK2 at a level beyond a threshold value they become deactivated and are removed from Zone 1 (TI_CK2_Threshold).
