# Supplementary material for: The Basic Immune Simulator: An agent-based model to study the interactions between innate and adaptive immunity
Source: Theor Biol Med Model. 2007 Sep 27;4:39. doi: 10.1186/1742-4682-4-39 (PMC2186321; doi:10.1186/1742-4682-4-39)
Supplement: Additional file 12 — T Cell agents (T2s) in Zone 1. A state diagram of the potential T2 behavioral sequences in Zone 1. [file 1742-4682-4-39-S12.pdf]

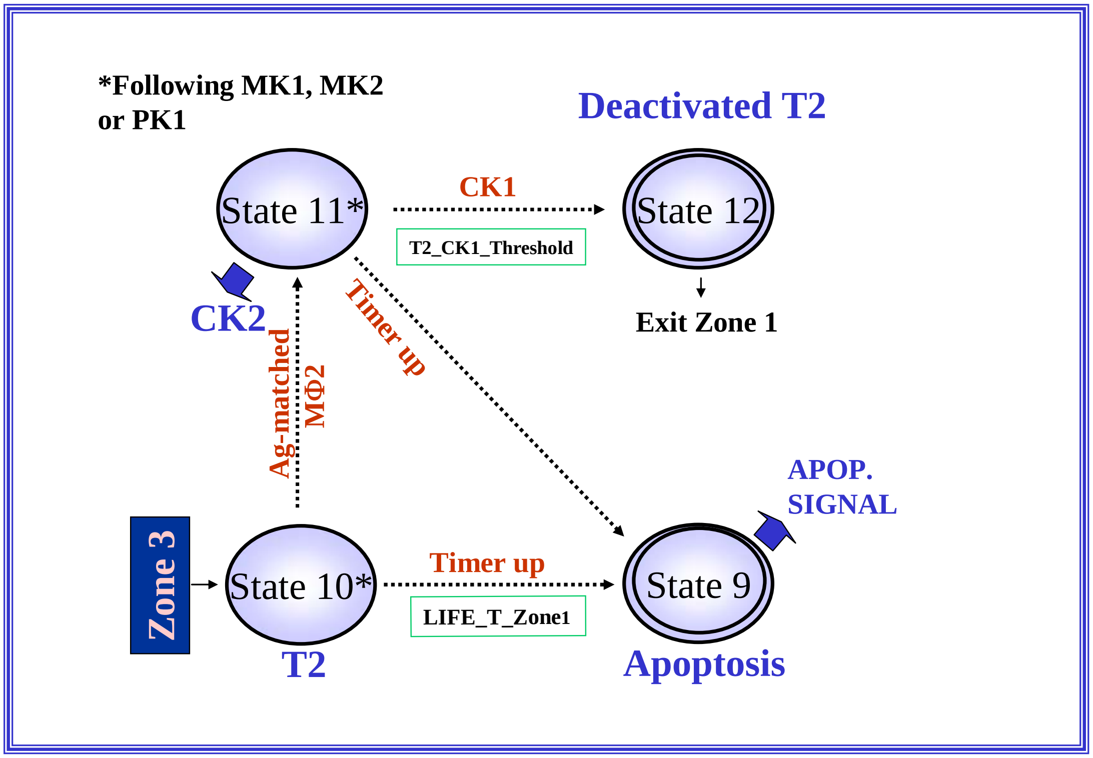

Additional file 12. State Diagram: T Cell agents (T2s) in Zone 1.
T2s enter Zone 1 and determine the levels of monokine-1 (MK1), MK2 and parenchymalkine-1 (PK1) in their immediate environment. They follow whichever signal is the greatest or move randomly. The T2s look for an antigen-matched Macrophage agent type 2 (MΦ2), and if they encounter one they produce cytokine-2 (CK2) [85]. If they sense a level of CK1 in their immediate environment that is above a threshold level they are deactivated and exit Zone 1 (<u>T2_CK1_Threshold</u>). Otherwise they remain in Zone 1 until they run out of time and undergo apoptosis (<u>LIFE_T_ZONE1</u>) [71].
