# Supplementary material for: The Basic Immune Simulator: An agent-based model to study the interactions between innate and adaptive immunity
Source: Theor Biol Med Model. 2007 Sep 27;4:39. doi: 10.1186/1742-4682-4-39 (PMC2186321; doi:10.1186/1742-4682-4-39)
Supplement: Additional file 13 — Cytotoxic T Lymphocyte agents (CTLs) in Zone 2. A state diagram of the potential CTL behavioral sequences in Zone 2. [file 1742-4682-4-39-S13.pdf]

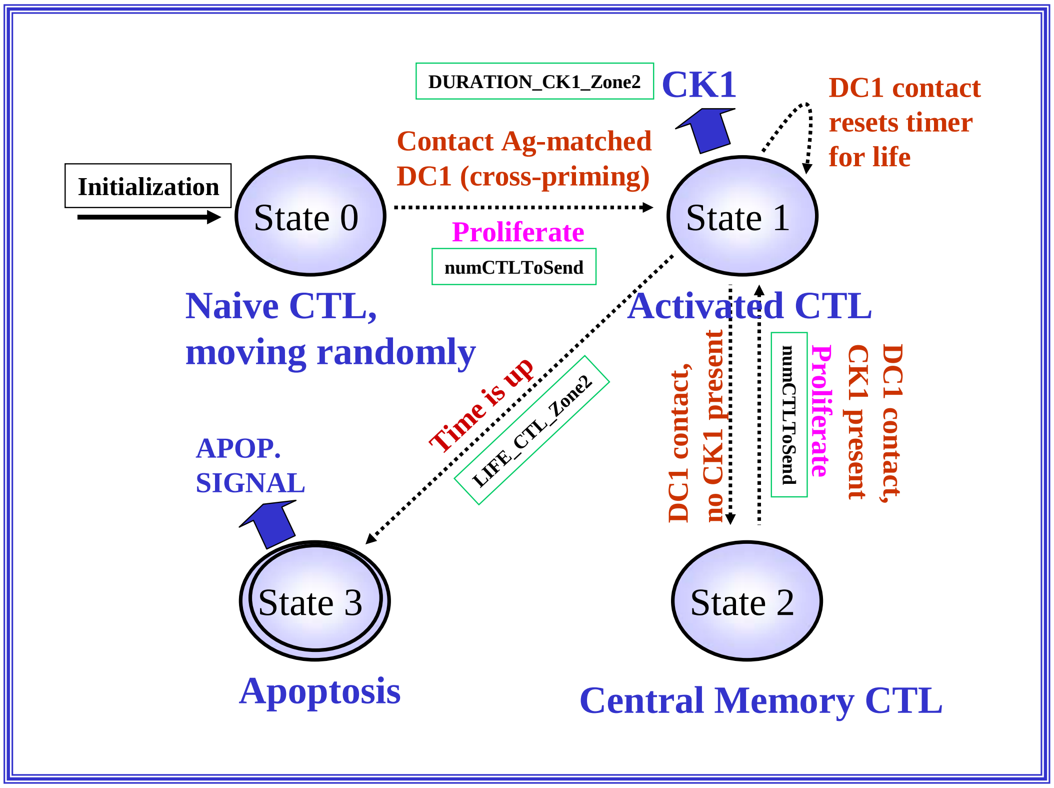

Additional file 13. State Diagram: Cytotoxic T Lymphocyte agents (CTLs) in Zone 2.

The CTLs begin in a resting state in Zone 2. The initial percentage of virus-specific CTLs is 0.4% (Additional file 17; PercentCTLAntiViral). They move randomly and wait for contact with an antigen-specific Dendritic Cell agent type 1 (DC1) in order to become activated (State 1). This process is referred to as "cross-priming" or "cross-presentation" [87]. This event also leads to cytokine production and proliferation of the CTLs (NumCTLToSend), and the progeny may migrate into Zone 3. Once the CTL is activated, subsequent contact with a DC1 will cause a transition to a memory CTL (State 2) if there is no cytokine-1 (CK1) present in the immediate environment [97]. Otherwise the contact extends the life of the CTL in State 1.
