# Supplementary material for: The Basic Immune Simulator: An agent-based model to study the interactions between innate and adaptive immunity
Source: Theor Biol Med Model. 2007 Sep 27;4:39. doi: 10.1186/1742-4682-4-39 (PMC2186321; doi:10.1186/1742-4682-4-39)
Supplement: Additional file 14 — Cytotoxic T Lymphocyte agents (CTLs) in Zone 1. A state diagram of the potential CTL behavioral sequences in Zone 1. [file 1742-4682-4-39-S14.pdf]

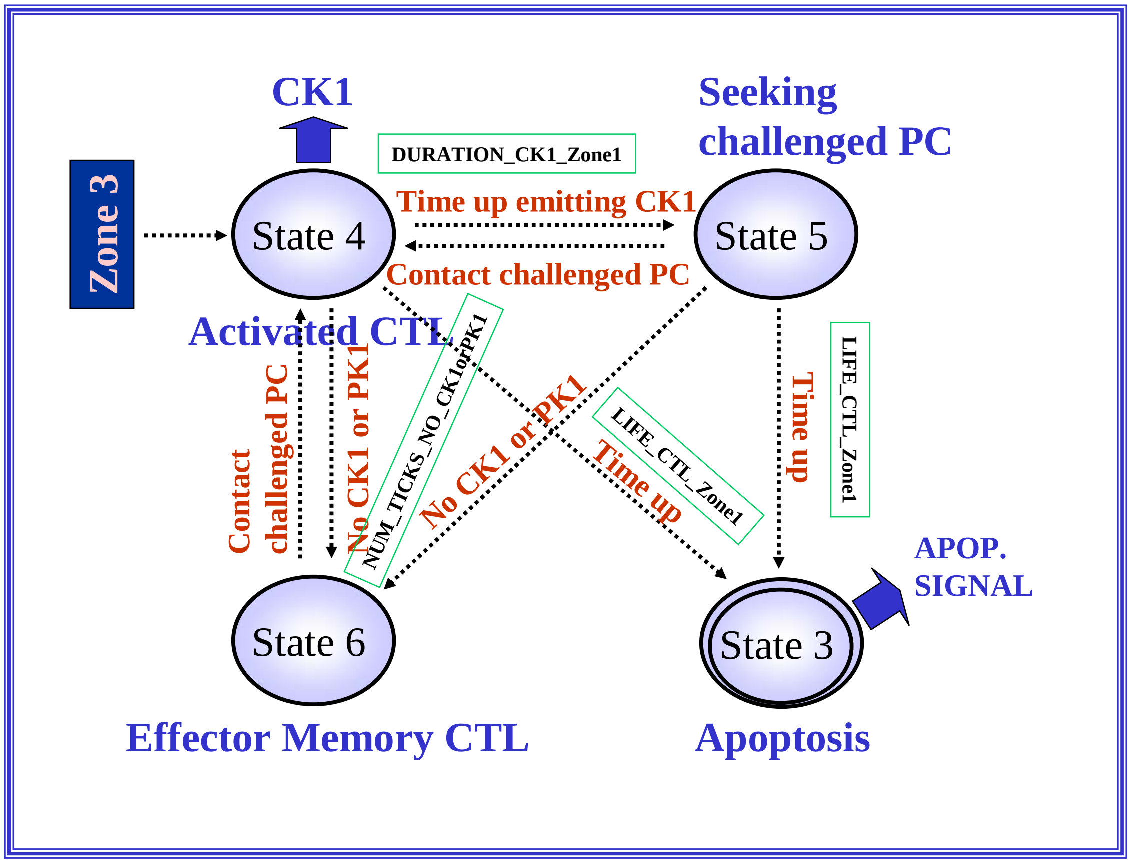

Additional file 14. State Diagram: Cytotoxic T Lymphocyte agents (CTLs) in Zone 1.

The CTLs migrate from Zone 3 into Zone 1 where they enter as activated, cytokine-1 (CK1)-producing agents. They produce CK1 for a finite period of time (DURATION_CK1_Zone1) that may be extended by contact with a virally infected Parenchymal Cell agent (PC). They continually sense CK1 and parenchymalkine-1 (PK1) in their immediate environment and follow PK1 to seek infected PCs, that they kill upon contact. In the absence of any cytokine for a defined period of time they become effector memory CTLs (State 6), a state that allows them to persist for a long period of time and from which they may become activated (back to State 4) by an encounter with a virally infected PC [97, 98].
