# Supplementary material for: The Basic Immune Simulator: An agent-based model to study the interactions between innate and adaptive immunity
Source: Theor Biol Med Model. 2007 Sep 27;4:39. doi: 10.1186/1742-4682-4-39 (PMC2186321; doi:10.1186/1742-4682-4-39)
Supplement: Additional file 16 — Portal agents in Zones 1, 2 and 3. A state diagram of the potential Portal agent behaviors in Zones 1, 2 and 3. [file 1742-4682-4-39-S16.pdf]

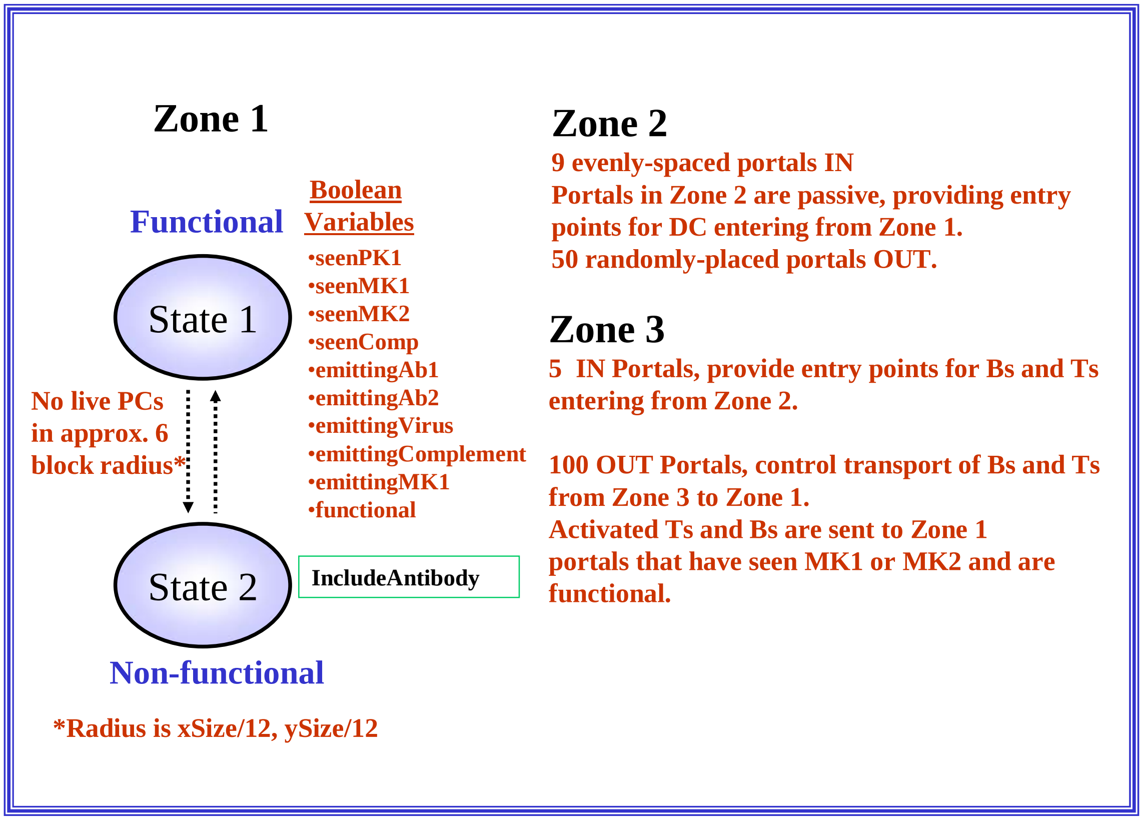

Additional file 16. State Diagram: Portal agents (Portals).

Portals are stationary agents present in all of the zones that control the migration of all of the other agent types that move from one zone to another. The Portals do not represent any immune cell type. They mark the locations in the zones that agents may use to migrate, abstractly representing the endothelium. Portals also mark the places that signal produced in one zone may diffuse through to enter another zone. Portals in all of the zones sense the signals that are present near to them and they record this information for inquiry by other agents.

There are 36 portals in Zone 1 that have two states, functional or non-functional, controlled by the state of the Parenchymal Cell agents (PCs) that surround them. If all of the PCs within a particular radius surrounding them have died, they are not functional (State 2). They return to a functional state when the PCs surrounding them are regenerated. Zone 2 contains portals that allow the migration of DCs into the zone, and the migration of the B Cell agents (Bs), T Cell agents (Ts) and Cytotoxic T Lymphocyte agents (CTLs) that have proliferated and then migrated out of Zone 2 into Zone 3.

Zone 3 has 5 portals that mark the locations that agents may enter, and 100 portals that mark the spaces that the randomly moving agents may exit from to migrate to Zone 1. The agents need only land on the coordinates with portals in Zone 3 for the transport to occur. Some agents have rules about the conditions of the portals in Zone 1 where they may enter. For some agents, certain signals must be present at the portal of entry in Zone 1 in order for them enter. Migration is delayed for agents that cannot find the proper conditions for entry into Zone 1.
