# Supplementary material for: The Basic Immune Simulator: An agent-based model to study the interactions between innate and adaptive immunity
Source: Theor Biol Med Model. 2007 Sep 27;4:39. doi: 10.1186/1742-4682-4-39 (PMC2186321; doi:10.1186/1742-4682-4-39)
Supplement: Additional file 17 — Input parameters for simulation runs. A table of all of the input parameters and the Zones that they affect. [file 1742-4682-4-39-S17.pdf]

Additional file 17 – Input parameters for simulation runs.

Parameter Name	Default Value	Notes
Ab1_Lysis_Threshold	100 signal units	Additional file 2
AllograftRadius	20 grid squares	NA
B1_Mem_CK1_Threshold	500 signal units	
B2_Mem_CK2_Threshold	500 signal units	
ChallengeXCoordinate	50 zone location	
ChallengeYCoordinate	50 zone location	
DC_Apop_Threshold	100 signal units	Additional file 3
DiffusionConstant	1.0	
DURATION_Ab1_Zone1	150 ticks	Additional file 7
DURATION_Ab2_Zone1	150 ticks	Additional file 7
DURATION_CK1_Zone1	25 ticks	Additional file 14
DURATION_CK1_Zone2	25 ticks	Additional files 10, 13
DURATION_CK2_Zone2	25 ticks	Additional file 10
DURATION_MK1_Zone1	25 ticks	Additional files 3, 5
DURATION_MK2_Zone1	25 ticks	Additional file 3, 5
DURATION_MK_Zone2	25 ticks	Additional file 4
DURATION_NK_CK1	25 ticks	Additional file 6
DURATION_Stressed	25 ticks	Additional file 2
DelayRegenerationTime	1 tick	Additional file 2
DuraEmitAbZ1	50 ticks	Additional file 9
DuraEmitAbZ3	50 ticks	Additional file 8
EvapRate	0.99	
Graft_DURATION_Stressed	5 ticks	NA
IncludeAntibody	true	Additional file 16
IncludeComplement	true	
IncrementOutputSignal	100 signal units	
InjuryRadius	5 grid squares	NA
LIFE_B_Zone1	25 ticks	Additional file 9
LIFE_B_Zone2	10 ticks	Additional file 7
LIFE_B_Zone3	25 ticks	Additional file 8
LIFE_CTL_Zone1	25 ticks	Additional file 14
LIFE_CTL_Zone2	25 ticks	Additional file 13
LIFE_DC_Zone1	50 ticks	Additional file 3
LIFE_DC_Zone2	100 ticks	Additional file 4
LIFE_GRAN_Zone1 (also used for Zone 3)	15 ticks	Additional file 15
LIFE_MO_Zone1	50 ticks	Additional file 5
LIFE_NK_Zone1	25 ticks	Additional file 6
LIFE_T_EXTRA	25 ticks	Additional file 11
LIFE_T_Zone1	20 ticks	Additional files 11, 12
LIFE_T_Zone2	13 ticks	
LIFE_T_Zone3	50 ticks	
LIMIT_NUM_Ts	12 agents	Additional file 4

Limit_Num_Tumor_Neighbors	28 agents	NA
MO1to2_Apop_Threshold	500 signal units	Additional file 5
NK_KILL_LIMIT	15 agents	Additional file 6
NUM_TICKS_NK_NO_KILL	15 ticks	Additional file 6
NUM_TICKS_NO_CK1orPK1	5 ticks	Additional file 14
NumB_ToSend	4 agents	Additional file 7
NumCTLToSend	1 agent	Additional file 13
NumDCToSend	1 agents	Additional file 3
NumDendriticAgents	50 agents	Number of DCs present at beginning of simulation run. Additional file 3
NumGranZ3denom	20 grid spaces	Controls the number of Gran in Zone3 at initialization. The number of grid spaces divided by this number is the number of Gran.
NumMoToSend	5 agents	Additional file 3
NumNKToSend	4 agents	Additional file 3,6
NumT1_ToSend	2 agents	Additional file 10
NumT2_ToSend	2 agents	Additional file 10
Num_Memory_B	0 agents	
Num_Memory_T	0 agents	
OutputSignal	1000 signal units	
Pause	-1 ticks	
PercentBAntiAllo	2 percent	NA
PercentBAntiTumor	2 percent	NA
PercentBAntiViral	2 percent	Percent of B's present in Zone 2 that can respond to the viral scenario.
PercentCTLAntiAllo	2 percent	NA
PercentCTLAntiTumor	0.4 percent	NA
PercentCTLAntiViral	0.4 percent	Percent of CTL's present in Zone 2 that can respond to the viral scenario.
PercentProInflammatory	95 percent	The percent of the DC's present initially that are DC1 (the remainder are DC2) Additional file 3
PercentTAntiAllo	2 percent	NA
PercentTAntiTumor	0.4 percent	NA
PercentTAntiViral	0.4 percent	Percent of the T's present in Zone 2 that can respond to the viral scenario.
Set_Graft	0 (false)	
Set_Injury	0 (false)	

Set_Tumor	0 (false)	
Set_ViralInfection	true	
StopSimulationAt	1000 ticks	Number of ticks for simulation run.
T1_CK2_Threshold	100 signal units	Additional file 11
T2_CK1_Threshold	100 signal units	Additional file 12
T_MAX_KILLS	10 agents	Additional file 11
Ticks_Tumor_Grows	15 ticks	NA
Too_Many_Tumor_Neighbors	64 agents	NA
Tumor_Mult_Freq	5 ticks	NA
Viral_Infection_Threshold	50 signal units	Additional file 2
World1XSize	100 grid squares	Zone 1 horizontal dimension
World1YSize	100 grid squares	Zone 1 vertical dimension
World2XSize	70 grid squares	Zone 2 horizontal dimension
World2YSize	70 grid squares	Zone 2 vertical dimension
World3XSize	70 grid squares	Zone 3 horizontal dimension
World3YSize	70 grid squares	Zone 3 vertical dimension

A list of all of parameters that have initial values that may be specified by the user. The notes indicate the figure where the parameter is incorporated into the rules. Most may be found in the figures in green boxes. Some of the parameters do not apply to simulation runs using the viral scenario (NA).
