# Supplementary material for: The Basic Immune Simulator: An agent-based model to study the interactions between innate and adaptive immunity
Source: Theor Biol Med Model. 2007 Sep 27;4:39. doi: 10.1186/1742-4682-4-39 (PMC2186321; doi:10.1186/1742-4682-4-39)
Supplement: Additional file 15 — Granulocyte agents in Zones 1 and 3. A state diagram of the potential Granulocyte agent behavioral sequences in Zones 1 and 3. [file 1742-4682-4-39-S15.pdf]

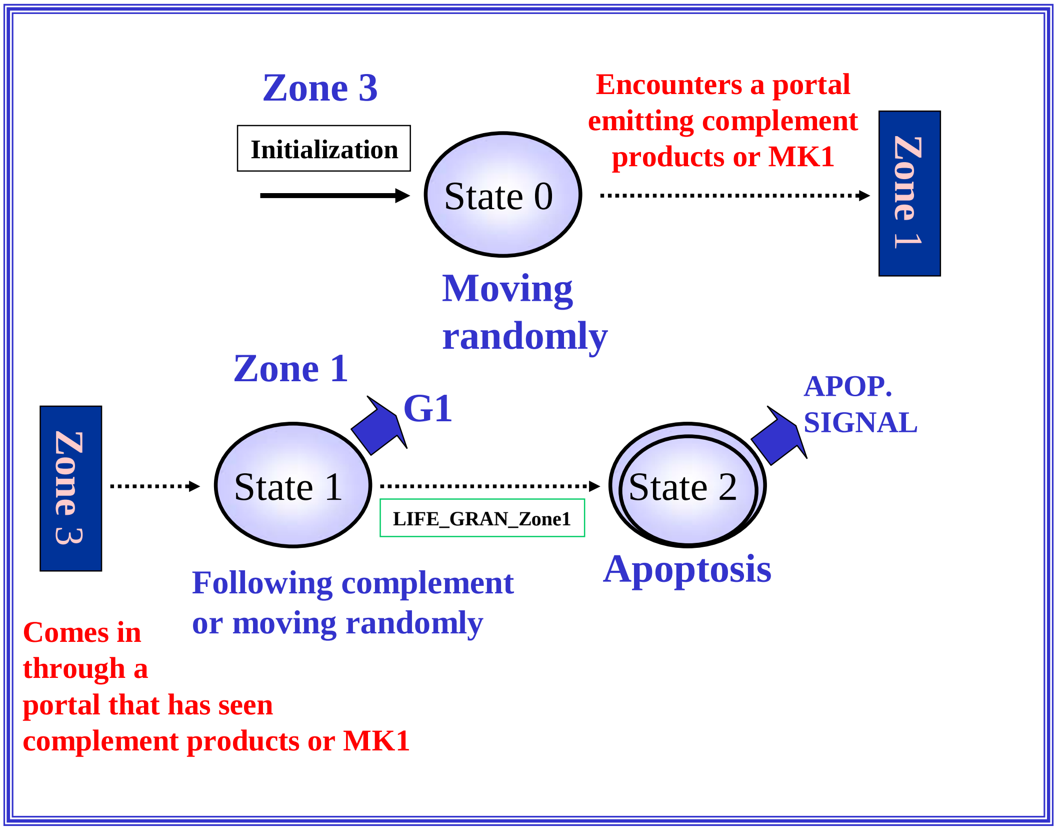

Additional file 15. State Diagram: Granulocyte agents in Zones 1 and 3.

Granulocyte agents begin in Zone 3, moving randomly until they detect a portal emitting complement products [70] or monokine-1 (MK1). At the detection of this signal they migrate into Zone 1 where they may follow the complement product signal or move randomly. They have a short lifetime in Zone 1 (LIFE_Gran_Zone1), during which they emit a degranulation signal that is lethal for any stressed Parenchymal Cell agent (PC) that encounters it [74].
